# Effect of Compaction on the Sound Transmission Loss of Automotive Textile Recycling

**DOI:** 10.3390/ma19204342

**Published:** 2026-10-15

**Authors:** Miroslav Badida, Miriama Pinosova, Miriam Andrejiova, Marek Moravec, Anna Badidová

**Affiliations:** 1 Department of Quality, Safety, and Environment, Faculty of Mechanical Engineering, Technical University of Košice, 042 00 Košice, Slovakia; miroslav.badida@tuke.sk (M.B.); miriama.pinosova@tuke.sk (M.P.); anna.badidova@tuke.sk (A.B.); 2 Department of Applied Mathematics and Informatics, Faculty of Mechanical Engineering, Technical University of Košice, 042 00 Košice, Slovakia; miriam.andrejiova@tuke.sk

**Keywords:** automotive textile waste, sound transmission loss, sound insulation, compaction pressure, material thickness, textile recycling

## Abstract

Automotive textile waste is a potential secondary raw material for acoustic insulation; however, the sound transmission loss (TL) of loose textile recyclate under controlled compaction remains insufficiently described. This study evaluates TL in loose, compacted, and compact forms. Loose fractions were tested without compaction and after compaction at 250, 500, and 750 kPa, while compact textile specimens served as references. Specimens 20, 45, and 70 mm thick were measured in a four-microphone impedance tube over 100–2500 Hz. Transmission coefficients, a relative transmission ratio, regression models, and dominance analysis were used to compare configurations and quantify the effects of frequency, thickness, and compaction pressure. The uncompacted fraction showed the lowest TL, increasing only from 4.20 to 6.27 dB as thickness increased from 20 to 70 mm. At 750 kPa, average TL increased from 14.98 dB at 20 mm to 36.34 dB at 70 mm. For loose fractions, the pressure–thickness interaction dominated TL (Std. beta = 0.764; *R*^2^ = 0.933), whereas compact specimens were governed mainly by the frequency–thickness interaction (Std. beta = 0.806; *R*^2^ = 0.976). Within the tested configurations, increasing compaction resulted in a pronounced reduction in the transmission of acoustic energy through the loose textile fraction, and at the highest compaction levels the TL values were comparable to or higher than those measured for the reference CT configurations.

## 1. Introduction

Fibrous and porous materials are an important group of acoustic materials used for noise reduction in technical and transportation applications. In automobiles, textiles and nonwoven structures are used primarily in floor carpets, luggage compartments, headliners, door panels, hood liners, and other components that affect interior noise and occupant comfort. Acoustic textiles in vehicles are intended to reduce airborne sound transmission, provide damping, and absorb sound [1]. Previous studies have also shown that both sound absorption and sound transmission loss (TL) depend on the function of the individual layers, material composition, fiber type and diameter, areal density, thickness, compression, density, porosity, airflow resistivity, and air permeability [2,3,4,5,6,7].

In addition to conventional fibrous insulation materials, increasing attention is being paid to recycled textile materials, including technical textiles from the automotive industry and end-of-life vehicles, which is also related to European requirements for their reuse, recycling, recovery, and the broader circularity of vehicles and end-of-life vehicle management [8,9]. Danihelová et al. [10,11] investigated STERED products, including Senizol AT XX2 TL60 panels made from recycled automotive technical textiles, and confirmed their suitability as insulation materials with high sound-absorbing properties. The European Commission has also reported on the practical use of STERED technology for processing carpets, upholstery, and production of cuts from automotive manufacturing [12]. The importance of material parameters—particularly thickness, areal density, density, porosity, layering, and internal fibrous structure—has also been confirmed by other studies of textile and nonwoven insulation materials, recycled polyester and PET nonwovens, and textile, apparel, and automotive waste suitable for the development of acoustic materials [13,14,15,16,17,18,19,20].

These findings are complemented by studies covering a broader range of recycled, natural, and synthetic fibrous materials, including recycled polyester fibers, such as PET fibers derived from plastic bottles [21]; recycled cotton and denim fibers from textile waste [7]; cotton/PET blends [22,23]; natural fibers, such as sheep wool, jute, and hemp [24,25]; and polypropylene-based synthetic nonwovens [26]. These studies confirm that the acoustic performance of fibrous materials results from a combination of material composition, processing method, internal structure, thickness, and frequency-dependent material behavior [27,28,29,30].

The available literature indicates that recycled textile materials have substantial potential for acoustic applications. However, most previous studies have focused primarily on finished boards, felts, layered or pressed structures, and nonwoven materials and have primarily evaluated their sound-absorption properties. Less attention has been paid to sound transmission loss (TL) in loose textile fractions derived from automotive textile recyclate, particularly under controlled variations in thickness and compaction. These parameters may affect bulk density, internal fiber arrangement, pore structure, and material permeability. It was hypothesized that increasing compaction pressure and specimen thickness would reduce acoustic-energy transmission and consequently increase TL in the loose textile fraction. The aim of this study was to experimentally assess the TL of automotive textile recyclate by comparing uncompacted and compacted loose textile fractions with compact textile specimens prepared from the same source material. Particular attention was given to the effects of specimen thickness, compaction pressure, and frequency on TL, the transmission coefficient, and the relative transmission behavior of the evaluated configurations.

## 2. Materials and Methods

The experimental study was designed to compare two forms of automotive textile recyclate prepared from the same source material: a loose textile fraction and compact textile specimens. For the loose-textile-fraction specimens, the effects of specimen thickness (*h*) and controlled compaction (*p*) were evaluated. From a physical perspective, compaction can be associated with changes in the bulk arrangement of the material and its bulk density, while accompanying changes in porosity and the internal fibrous structure may also occur. However, porosity, airflow resistance, pore-size distribution, and the internal arrangement of the textile structure were not directly measured in this study. Compact textile specimens (CT) served as reference specimens with a cohesive, compressed fibrous structure and were used for comparison with specimens prepared from the loose textile fraction. Transmission loss (TL) was measured using a BSWA SW433 impedance tube (BSWA Technology Co., Ltd., Beijing, China) in a four-microphone configuration over the frequency range 100–2500 Hz. Five repeated measurements, which were performed for each experimental configuration, formed the basis for the subsequent statistical evaluation.

### 2.1. Investigated Material and Test Specimens

The automotive textile recyclate used in this study was supplied by the industrial partner PR Krajné, s.r.o. (STERED^®^, Krajné, Slovakia), which specializes in the material recovery of technical textiles from the automotive industry. According to the VÚCHV certificate [31], the STERED fiber blend is characterized as a material containing 90–100% man-made fibers, including polyester, polyamide, polypropylene, m-aramid, viscose, polyurethane, and other components, while the proportion of natural fibers ranges from 0 to 10%. The general characteristics of the fiber types used in STERED products are further described in VÚCHV Report No. 03/2012 [32]. The percentage distribution of the individual polymer components in the specific material batch used in this study was not determined separately by analytical methods.

**Figure 1:**
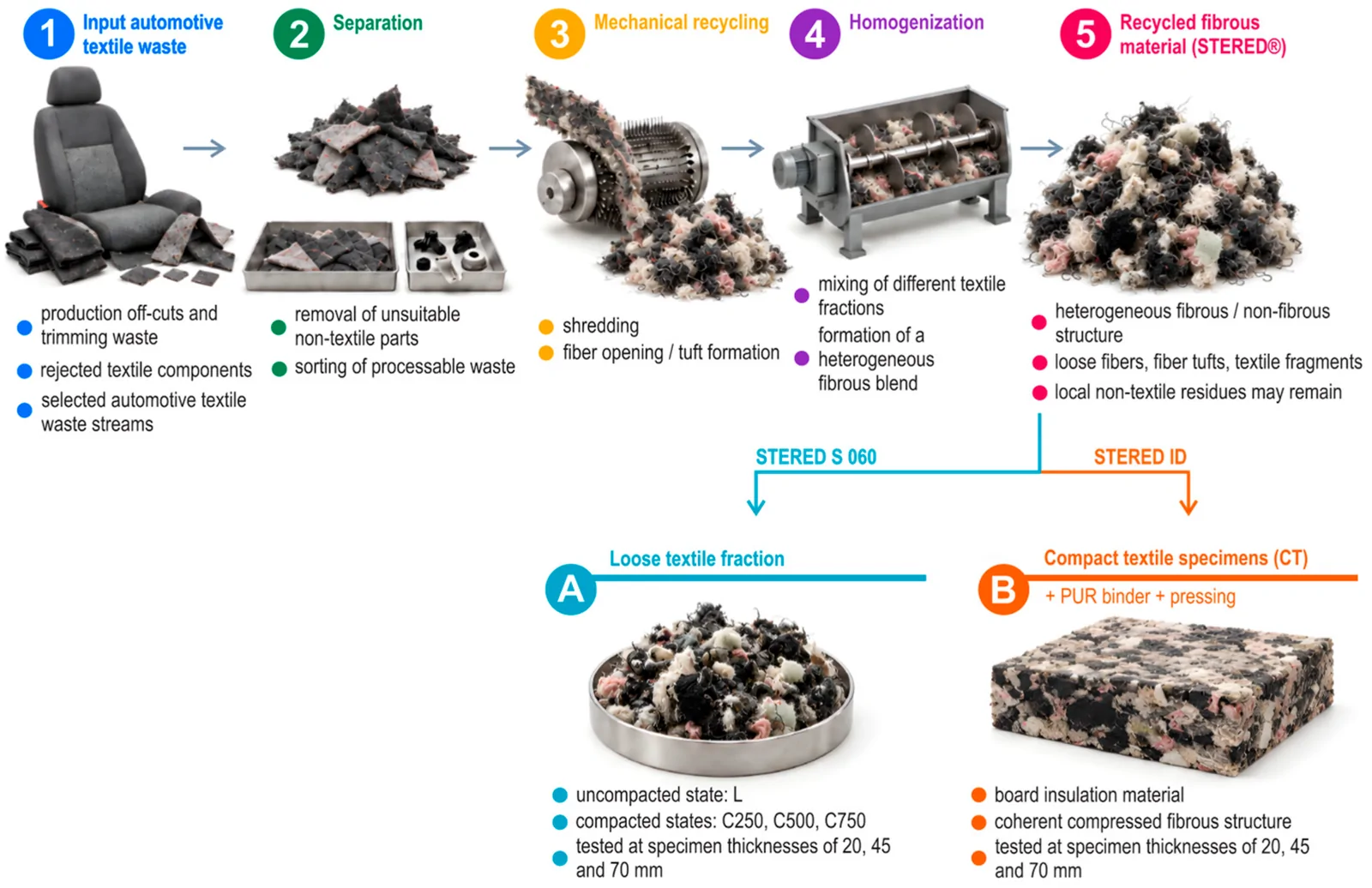
Schematic representation of the mechanical recycling process is used to convert automotive technical textile waste into the recycled fibrous material evaluated in this study.

According to the supplier’s documentation, the recycled material originates from technical textiles used in the automotive industry, particularly from production cut-offs, trimmings, and other textile residues generated during the manufacture of automotive components, as well as from textile parts recovered during the processing of end-of-life vehicles [33]. The processed technical textiles may originate, for example, from floor carpets, upholstery, covering fabrics, insulation materials, and other interior components. The exact proportion of the individual types of input textiles in the specific material batch used in this study was not documented. The mechanical recycling process used to convert automotive textile waste into the recycled fibrous material evaluated in this study is schematically illustrated in Figure 1.

Mechanical processing of automotive textile components produces a loose heterogeneous textile fraction composed of fibers, fibrous agglomerates, and fragments of the original textile structures. The material may also contain residual non-textile components, such as plastics or polyurethane. Before preparation of the test specimens, the mechanically processed textile fraction was thoroughly mixed to achieve the most uniform possible distribution of its individual components. All tested loose-textile-fraction configurations were prepared from the same homogenized material batch. The acoustic properties of fibrous and porous materials depend on their structural and material characteristics, including thickness, bulk density, and parameters associated with airflow through the pore structure [6,7,34,35]. Therefore, specimen thickness and compaction state were selected as the main experimental variables in this study.

**Figure 2:**
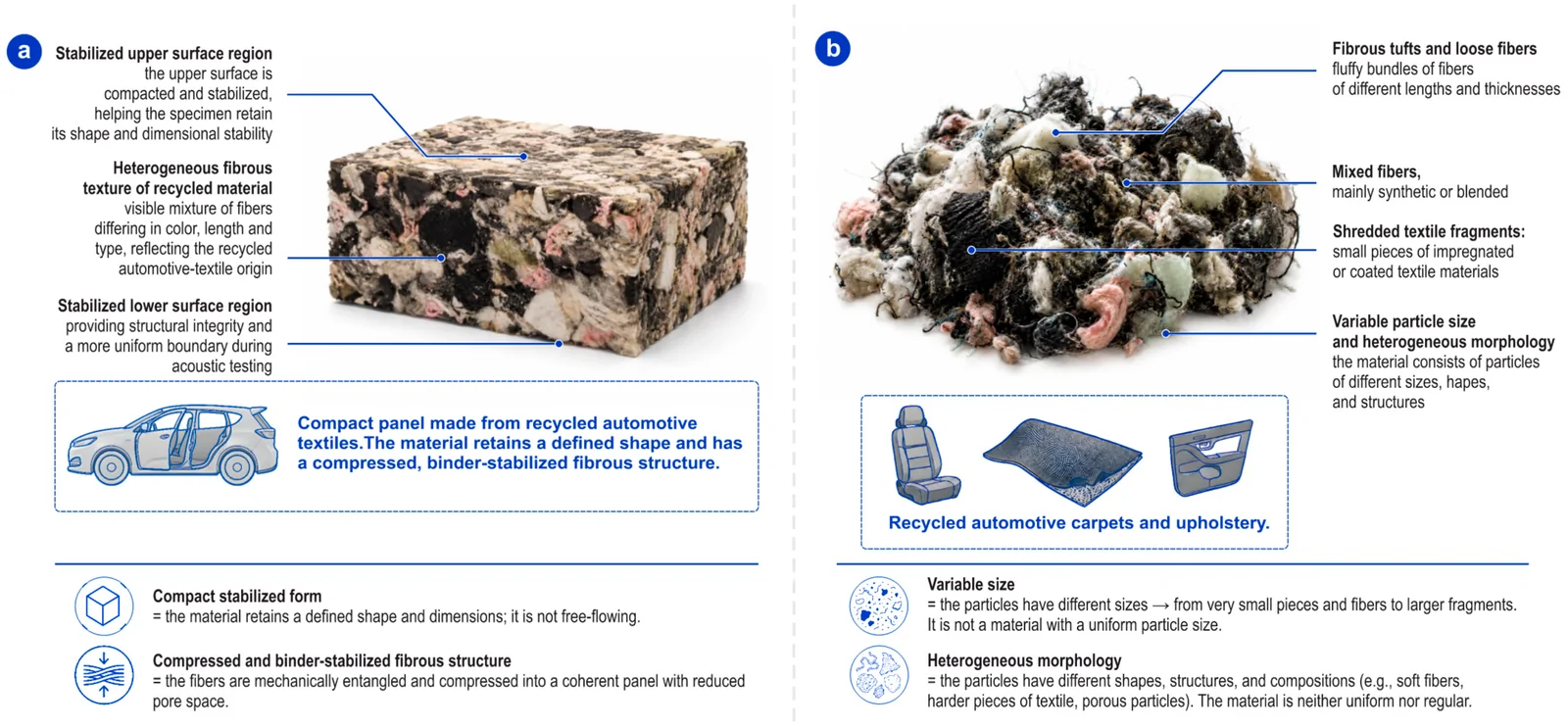
Evaluated forms of automotive textile recyclate: (**a**) Compact textile specimen prepared from board insulation material; (**b**) loose textile fraction consisting of fibrous tufts, loose fibers and shredded textile fragments.

In this study, the automotive textile recyclate was evaluated in two material forms: a loose textile fraction and compact textile specimens prepared from board insulation material. The loose textile fraction was evaluated without compaction (*p* = 0 kPa; code L) and after compaction at nominal pressures of *p* = 250, 500, and 750 kPa (codes C250, C500, and C750). The compact textile specimens were evaluated without additional compaction (code CT). A visual comparison of the compact form and the loose textile fraction is shown in Figure 2. The codes used to identify the evaluated forms of automotive textile recyclate are listed in Table 1.

The compact textile specimens were prepared from board insulation material using automotive textile recyclate. The board insulation material was manufactured from a homogenized fibrous mixture stabilized with a polyurethane (PUR) binder, which ensured the cohesion and mechanical stability of the compact fibrous structure. The available manufacturer documentation specifies the use of a PUR binder but does not provide its exact mass fraction or detailed technological parameters of the board manufacturing process. Circular specimens with thicknesses of *h* = 20, 45, and 70 mm and a diameter of *d* = 60 mm were prepared from this material. The specimens were evaluated without additional compaction and are labelled as CT throughout the remainder of the manuscript. The supplementary material characteristics of the board insulation material, provided by PR Krajné, s.r.o. (STERED^®^, Krajné, Slovakia), are presented in Table 2. These parameters represent the declared properties of the board insulation material and provide a more detailed material description of the compact textile specimens.

The bulk density of 200 kg·m^−3^ reported in Table 2 represents the manufacturer-declared characteristic of the board insulation material. In contrast, the bulk density values of the individual CT specimens reported in Table 3 were calculated from the measured specimen mass and its geometric volume. These values therefore characterize the specific test specimens used in the acoustic measurements rather than the nominal property of the board material. The loose textile fraction consisted of an irregular mixture of textile particles, fibrous tufts, and loose fibers. Unlike the board insulation material, it did not form a cohesive compact structure; its internal arrangement therefore depended on the manner of placement, the amount of material inserted, specimen thickness, and degree of compaction. Specimens made from the loose textile fraction were evaluated at thicknesses of *h* = 20, 45, and 70 mm. The uncompacted state was designated L, whereas the compacted states were designated C250, C500, and C750 based on the nominal compaction pressures of *p* = 250, 500, and 750 kPa (see Table 3). Compaction could alter bulk density, internal fiber arrangement, pore size, and contact between textile particles, that is, parameters associated with acoustic-energy transmission through the test specimen. A detailed description of the test cassette and the device used to prepare compacted states is provided in Section 2.2.

In addition to bulk density (ρ), the areal mass (*m_s_*) was determined for each experimental configuration and calculated as the ratio of specimen mass (*m*) to its cross-sectional area (*A*), *m_s_* = *m*/*A*. For the loose-textile-fraction specimens, the cross-sectional area was determined from a diameter of 49 mm, and for the compact textile CT specimens from a diameter of 60 mm. The areal-mass values of the individual configurations are given in Table 3.

Compaction pressure *p* and specimen thickness *h* are controlled experimental factors. However, changes in these factors are also accompanied by changes in specimen mass, bulk density, and areal mass; therefore, these parameters cannot be interpreted as completely independent physical characteristics of the material.

Based on this designation, the experimental configurations were defined by the combination of material form, compaction state, and specimen thickness. The number following the hyphen in the configuration code denotes the specimen thickness *h* in millimeters. For example, the code C500-45 denotes a compacted textile fraction at a nominal compaction pressure of *p* = 500 kPa and a thickness of *h* = 45 mm. The subsequent TL evaluation was based on comparisons of the uncompacted loose textile fraction (L, *p* = 0 kPa), its compacted states (C250, C500, and C750), and compact textile specimens (CT) prepared at three thicknesses. Basic data for the prepared experimental configurations used in the TL evaluation are presented in Table 3.

### 2.2. Test Cassette and Compaction Device

**Figure 3:**
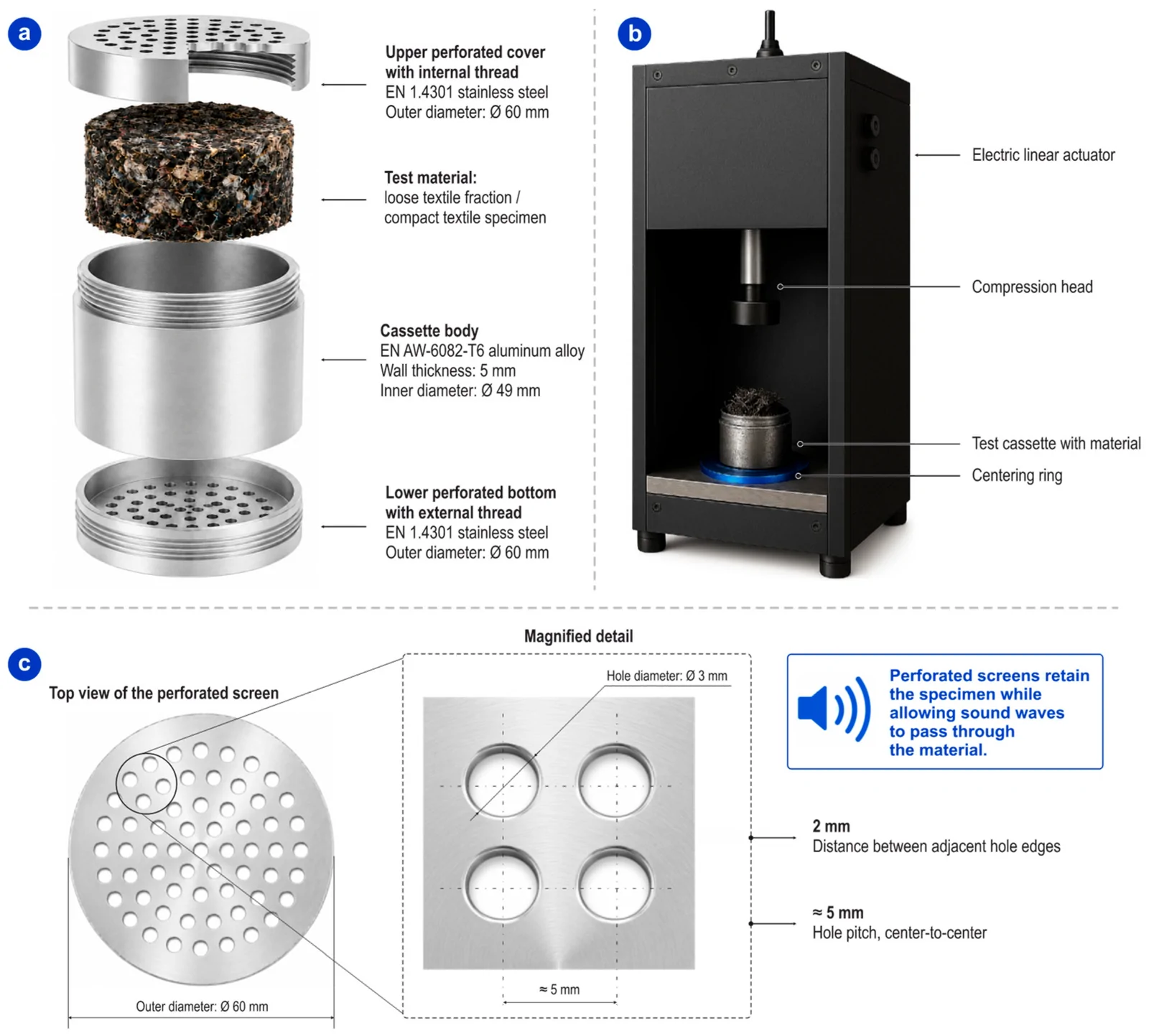
Test cassette and compaction device used for specimen preparation: (**a**) exploded view of the cassette with perforated cover, test material, cassette body and perforated bottom; (**b**) cassette positioned in the compaction device; (**c**) geometry of the perforated stainless-steel screen.

A test cassette based on Patent SK 289324 B6 [36,37] was used to prepare and measure specimens made from the loose textile fraction. Because of the non-cohesive structure of the material, the cassette Figure 3a was used to maintain the defined specimen thickness and compaction state during preparation, handling, and measurement in the impedance tube. The cassette body, with a wall thickness of 5.5 mm and an internal diameter of *d_i_* = 49 mm, was manufactured from Al 6082-T6 aluminum alloy. The inner diameter of the cassette $d_{i}=49\;mm$ corresponded to the diameter of the tested loose-textile-fraction specimen. In contrast, the compact textile specimens (CT) had a diameter of 60 mm and were inserted directly into the measuring section of the impedance tube without using the test cassette. The loose textile fraction was enclosed in the cassette between a lower perforated base and an upper perforated cover made of 1.4301 stainless steel with a regular circular perforation pattern comprising 3-mm-diameter holes at a center-to-center spacing of 5 mm. In this study, specimens with thicknesses of *h* = 20, 45, and 70 mm were prepared to evaluate the effects of specimen thickness *h* and compaction pressure *p* on TL. After the cassette had been filled and the material adjusted to the required thickness, it was closed using the upper perforated cover, which stabilized the material during handling and subsequent insertion into the impedance tube. A controlled-filling device based on Patent SK 289359 B6 [38,39] was used to compact the material Figure 3b. The cassette containing the loose textile fraction was positioned in a centering ring on a scale, thereby ensuring coaxial alignment with the electric linear actuator. During preparation of the compacted specimens, the loose textile fraction was added to the test cassette progressively in smaller quantities and continuously compressed using an electric linear actuator. Material was added and compacted in this manner until the required specimen thickness and corresponding nominal load were reached. The applied load was continuously monitored during compaction. Three load levels were applied: 50, 100, and 150 kg. For a cassette with an internal diameter of *d_i_* = 49 mm, these load levels were defined in this study as nominal compaction pressures of *p* = 250, 500, and 750 kPa. This procedure produced three compacted states, designated C250, C500, and C750. After the target condition was reached, the specimen was maintained under the corresponding load for 60 s. The load was then released, and the upper perforated cover of the cassette was secured so that the prescribed specimen thickness and material arrangement were maintained during handling and acoustic measurement. Only slight elastic recovery of the material was observed after unloading; this was not quantified as a separate experimental parameter. The actuator travel speed was not recorded as a separate quantitative parameter.

The uncompacted state (L) was prepared by loosely filling the cassette without additional mechanical compression.

The combination of the test cassette and controlled-filling device enabled specimens to be prepared with a defined thickness and different degrees of compaction. The resulting experimental configurations were used to systematically evaluate the effects of thickness, bulk density, and internal material arrangement on TL.

### 2.3. Transmission Loss Measurement Using an Impedance Tube

**Figure 4:**
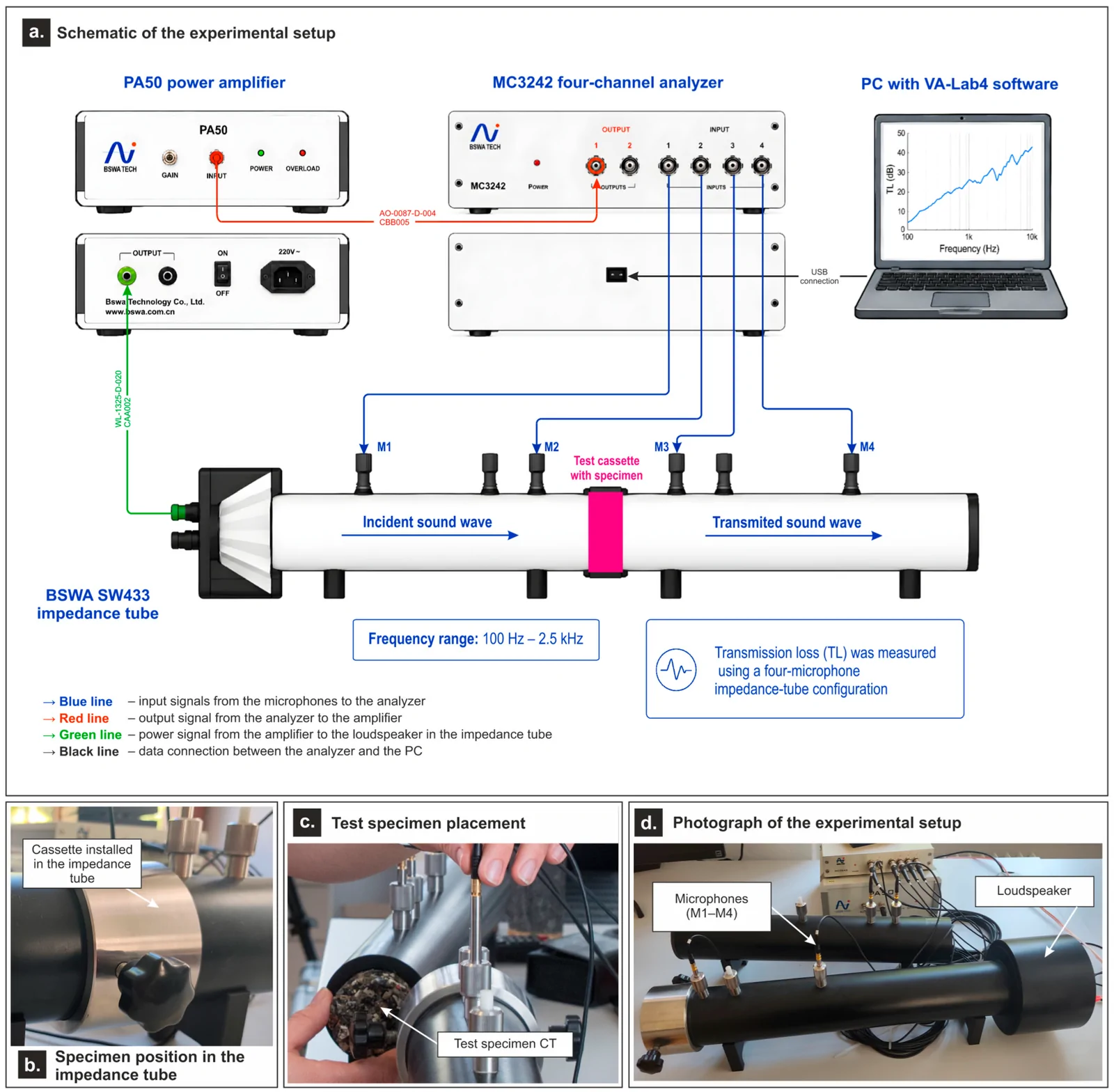
Experimental setup for transmission loss measurement using the BSWA SW433 impedance tube in a four-microphone configuration.

Transmission loss (TL) was measured using a BSWA SW433 impedance tube in a four-microphone configuration. The measurement system consisted of the impedance tube, four microphones, an MC3242 four-channel analyzer (BSWA Technology Co., Ltd., Beijing, China) with a frequency range 0–20 kHz, a 50-W PA50 amplifier (BSWA Technology Co., Ltd., Beijing, China) for driving the loudspeaker in the tube, a computer running VA-Lab4 software (version 6.0), and the associated cabling. The measurement was performed according to the measurement procedure specified by the equipment manufacturer, which is based on the transfer-function principle used in STN EN ISO 10534-2 [40]. Detailed technical parameters of the measurement configuration, including microphone spacing, the relevant frequency ranges, the calibration procedure, and the tube termination conditions, are provided in the cited manufacturer’s technical documentation and the relevant standard [40,41]. One specimen was prepared for each experimental configuration. Five consecutive technical measurements were performed on each specimen without removing or reinserting it into the impedance tube, thereby maintaining the same specimen position and configuration throughout the measurement series. These technical measurements characterized repeatability under unchanged specimen conditions rather than independent replication. In total, 15 specimens were evaluated: 12 loose-textile-fraction specimens and 3 compact CT specimens. No independent replicate specimens were prepared for the individual configurations. A schematic of the impedance-tube measurement system used to evaluate TL is shown in Figure 4.

To assess the effect of using the test cassette and reducing the diameter of the tested specimen, a control measurement was performed on a solid specimen. The same specimen was first measured at its original diameter of 60 mm directly in the impedance tube and was then trimmed to a diameter of 49 mm, inserted into the test cassette, and measured again. The difference between the results of the two measurements was approximately 1–1.5 dB in the individual frequency bands.

The measurement was based on the transfer-matrix approach used to determine normal-incidence sound transmission loss [42]. The measurement system also used the transfer-function principle between microphones, which underlies impedance-tube measurements of material acoustic properties under STN EN ISO 10534-2 (730537), the standard in force when the measurements were performed [40], and was configured in accordance with the manufacturer’s technical documentation [41]. The output parameter was TL (dB), which expresses the logarithmic ratio between the acoustic power incident on the front surface of the specimen and the power transmitted through the tested material. A higher TL value indicates a lower fraction of acoustic power transmitted through the specimen and therefore greater sound-insulation performance. Transmission loss was expressed as:

(1) $$TL=10{log}_{10}(W_{i}/W_{t})$$

where $W_{i}$ is the acoustic power incident on the test specimen and $W_{t}$ is the acoustic power transmitted through the specimen.

Measurements were conducted under stable laboratory conditions: the air temperature was 22.5 ± 1 °C, relative humidity was 48 ± 5%, and atmospheric pressure was 986.07 hPa. TL was measured over the frequency range 100–2500 Hz. All experimental configurations prepared following the procedures described in Section 2.1 and Section 2.2 were evaluated, namely the uncompacted loose textile fraction (L), the compacted states (C250, C500, and C750), and the compact textile specimens (CT). The TL evaluation focused primarily on the effects of frequency, specimen thickness, and material compaction on the transmission loss of the tested specimens.

### 2.4. Data Processing and Statistical Evaluation

The measured TL values were processed for each experimental configuration defined by material state and specimen thickness. For each experimental configuration, five technical replicates were performed on the same prepared specimen. At each evaluated frequency, the mean TL value and standard deviation were calculated from these measurements. The technical replicates were used to characterize measurement repeatability under an unchanged specimen configuration and did not represent independent experimental replicates. The mean TL values calculated from the five technical replicates were used in the subsequent regression analysis.

In the tables and configuration codes, specimen thickness is denoted by *h* and reported in millimeters because this notation corresponds directly to the experimental specimen codes. For the statistical evaluation, thickness was used as a numerical variable in centimeters, with *h_s_* = *h*/10. For regression modeling, the thickness values were converted to meters, namely 0.020, 0.045, and 0.070 m, so that the regression coefficients were expressed in SI units [43,44,45].

The statistical evaluation focused on assessing the effects of frequency *f*, specimen thickness *h_s_*, and compaction pressure *p* on TL. For the loose textile fraction, the effect of compaction was evaluated by comparing the uncompacted state L with the compacted states C250, C500, and C750. Compact textile specimens (CT) were evaluated separately and then compared with specimens made from the loose textile fraction at the same thickness. Bulk density ρ and areal mass *m_s_* were used as supplementary material parameters characterizing the individual experimental configurations.

To assess and compare the measured transmission-loss values, the following quantities characterizing the acoustic properties of the investigated specimens were defined: transmission loss at frequency *f* $\left(TL_{f}\right)$, transmission coefficient at frequency *f* ${(\tau }_{f})$, average transmission coefficient $\left(\tau_{avg}\right)$, average transmission loss $\left(TL_{avg}\right)$, and relative transmission ratio $\left(R{TR}_{f}\right)$.

Transmission loss (TL, dB) is defined as the logarithmic ratio of the incident and transmitted sound waves, expressed in terms of acoustic power or intensity. The relationship for transmission loss, equivalently expressed using the transmission coefficient at frequency *f*, can be written as follows:

(2) $${TL}_{f}=-10{log}_{10}(\tau_{f})$$

where $\tau_{f}$ represents the fraction of acoustic power transmitted through the test specimen at frequency *f*.

Because TL values are expressed in decibels, they had to be converted to linear values before further summation or averaging. The transmission coefficient was then calculated from the measured $TL_{f}$ values using:

(3) $$\tau_{f}=10^{-{TL}_{f}/10}$$

where $TL_{f}$ is the transmission loss at frequency *f*.

The transmission coefficient *τ_f_* represents the ratio of acoustic power transmitted through the specimen to the incident power. A value of *τ_f_* = 1 (100%) corresponds to *TL_f_* = 0 dB, *τ_f_* = 0.5 (50%) to approximately 3 dB, *τ_f_* = 0.1 (10%) to 10 dB, and *τ_f_* = 0.01 (1%) to 20 dB. The reciprocal value 1/*τ_f_* = *W_i_*/*W_t_* represents the ratio of incident to transmitted acoustic power, with *TL_f_* = 10log10(1/*τ_f_*). The energy-based, or linear, mean transmission coefficient $\tau_{avg}$ over the frequency range 100–2500 Hz was calculated as:

(4) $$\tau_{avg}=\frac{1}{n}\sum_{i=1}^{n}\tau_{f_{i}}$$

where n is the number of evaluated frequency bands. For each evaluated frequency, the mean *TL_f_* value was first calculated from the five technical repetitions. From this value, the linear transmission coefficient *τ_f_* was calculated according to Equation (3). The value of *τ_avg_* was then determined as the arithmetic mean of *τ_f_* over *n* = 15 one-third-octave bands in the range 100–2500 Hz, with equal weight assigned to each band.

The average transmission loss was subsequently obtained by converting the result back to the decibel scale:

(5) $${TL}_{avg}=-10{log}_{10}(\tau_{avg})$$

This relationship converts the average transmission coefficient $\tau_{avg}$ back to the average transmission loss expressed in decibels. Because averaging is performed on the linear energy scale, the resulting *TL_avg_* is influenced more strongly by frequency bands with lower TL values. Therefore, in this study *TL_avg_* is used solely as a comparative indicator of the evaluated experimental configurations and does not represent a standardized single-number rating such as *R_w_*.

To compare acoustic-energy transmission between two evaluated specimens, designated specimen A and specimen B, the relative transmission ratio $\left(R{TR}_{f}\right)$ was used:

(6) $${RTR}_{f}=\tau_{f,A}/\tau_{f,B}$$

where $\tau_{f,A}$ is the power transmission coefficient of specimen A at frequency *f*, and $\tau_{f,B}$ is the power transmission coefficient of specimen B at the same frequency. This ratio indicates how many times greater or lower the acoustic-energy transmission through one specimen is relative to the other. A value of $R{TR}_{f}>1$ indicates greater acoustic-energy transmission through specimen A than through specimen B, corresponding to a lower TL value for specimen A; $R{TR}_{f}<1$ indicates lower transmission through specimen A than through specimen B, corresponding to a higher TL value for specimen A; and $R{TR}_{f}\approx 1$ indicates comparable transmission behavior of the two specimens.

Regression analysis was used to assess the relationship between TL values and selected specimen parameters. The dependent variable was TL or $TL_{avg}$, whereas the independent variables considered were primarily frequency *f*, specimen thickness *h_s_*, and compaction pressure *p*. The general form of the multiple linear regression model can be written as:

(7) $$Y=\beta_{0}+\beta_{1}\cdot X_{1}+\beta_{2}\cdot X_{2}+\cdots +\beta_{k}\cdot X_{k}+\varepsilon$$

where Y is the dependent variable, $X_{1},X_{2},\ldots ,X_{k}$ are the independent variables, $\beta_{0}$ is the intercept, $\beta_{1},\beta_{2},\ldots ,\beta_{k}$ are the regression coefficients, and ε is the random model error.

The overall statistical significance of the regression models was evaluated using the *F*-test, and the significance of the individual regression coefficients was evaluated using *t*-tests [46,47]. The results were assessed at the significance level α=0.05. The explanatory power of the model was evaluated using the coefficient of determination $R^{2}$ and the adjusted coefficient of determination (adjusted $R^{2}$); higher values of these measures indicated better agreement between the model and the experimental data. Standardized regression coefficients (Std. *β*) were used to compare the relative importance of the individual input variables.

Linear regression analysis, including the calculation of standardized regression coefficients, was performed using the stats (v4.5.2) and lm.beta (v1.7-0) packages. The regression-model assumptions were evaluated by testing residual normality, constancy of residual variance, and multicollinearity between the independent variables. Multicollinearity was assessed using the variance inflation factor (VIF) calculated with the car package (v3.1-2). VIF values below 10 were considered acceptable for the regression models used in this study. When model assumptions were violated, robust standard errors were calculated using the lmtest (v0.9-40) and sandwich (v3.1-1) packages.

TL values obtained at the individual frequencies from the same specimen do not represent independent experimental units. The regression models are therefore interpreted as empirical models describing the relationships between TL and the investigated variables within the evaluated frequency range of 100–2500 Hz and the tested experimental configurations.

The out-of-specimen predictive performance of Models I and II was evaluated using leave-one-specimen-out cross-validation (LOSO-CV). In each fold, one physical loose-textile-fraction specimen, including all 15 band-wise mean TL values, was excluded. The regression model was refitted using the remaining 11 specimens and was then used to predict TL for the excluded specimen. This procedure was repeated for all 12 loose-textile-fraction specimens. Thus, no frequency-band observation from the specimen being predicted was included in model fitting in the corresponding fold. Cross-validated predictive performance was evaluated using the cross-validated coefficient of determination (R^2^_CV_), root mean square error (RMSE), mean absolute error (MAE), mean prediction error (bias), and the Pearson correlation coefficient (*r*) between measured and cross-validated predicted TL values. Model III was not subjected to specimen-level cross-validation because only three physical CT specimens were available, which was considered insufficient for a reliable assessment of out-of-specimen predictive performance.

Dominance analysis, used to quantify the relative contribution of individual variables to the explained variability in TL, was performed using the dominanceanalysis package (v2.1.1). The results and response surfaces were visualized using the ggplot2 package (v4.0.3). Additional graphical outputs depicting the modeled relationships were created in OriginPro 2019b.

The overall visual concept and composition of Figure 1, Figure 2 and Figure 3 were developed by the authors. ChatGPT (OpenAI, model GPT-5.6 Sol), including its AI-based image generation capabilities, was used to generate and modify selected illustrative elements. These elements were subsequently manually edited, redrawn where necessary, and incorporated into the final figures using CorelDRAW 2020 (version 22.0.0.412). The final graphical processing and composition of all three figures were carried out by the authors.

## 3. Results

This section evaluates the effects of selected specimen parameters on their ability to limit sound transmission. The variables considered were:

1. material form (uncompacted loose textile fraction L, compacted loose textile fractions C250, C500, and C750, and compact textile specimens CT),
2. specimen thickness (20, 45, and 70 mm; 2, 4.5, and 7 cm in the statistical evaluation),
3. compaction pressure (*p* = 0, 250, 500, and 750 kPa),
4. sound frequency (100–2500 Hz).

The results compare the transmission-loss values and transmission coefficients of loose-textile-fraction specimens at different compaction levels with those of compact textile specimens. The analysis focuses on the effects of specimen thickness, compaction pressure, and frequency on transmission loss and acoustic-energy transmission. The results make it possible to assess the sound-insulation performance of automotive textile recyclate in uncompacted, compacted, and compact forms and to identify the configurations that produce the highest transmission-loss values.

### 3.1. Definition of Variables

The automotive textile recyclate evaluated in this study was tested in the forms of an uncompacted loose fraction (L), compacted loose fractions (C250, C500, and C750), and compact textile specimens (CT). For the evaluation of transmission loss, the principal parameters were material form, specimen thickness, compaction pressure, areal mass, bulk density, and sound frequency because these parameters directly affect the material’s ability to limit acoustic-energy transmission. Table 4 provides an overview of the symbols and parameters used to characterize the investigated automotive-textile-recyclate specimens.

### 3.2. Transmission Loss

Transmission loss TL was evaluated as the primary indicator of the sound-insulation performance of the automotive textile recyclate as a function of frequency, compaction pressure, and specimen thickness.

**Figure 5:**
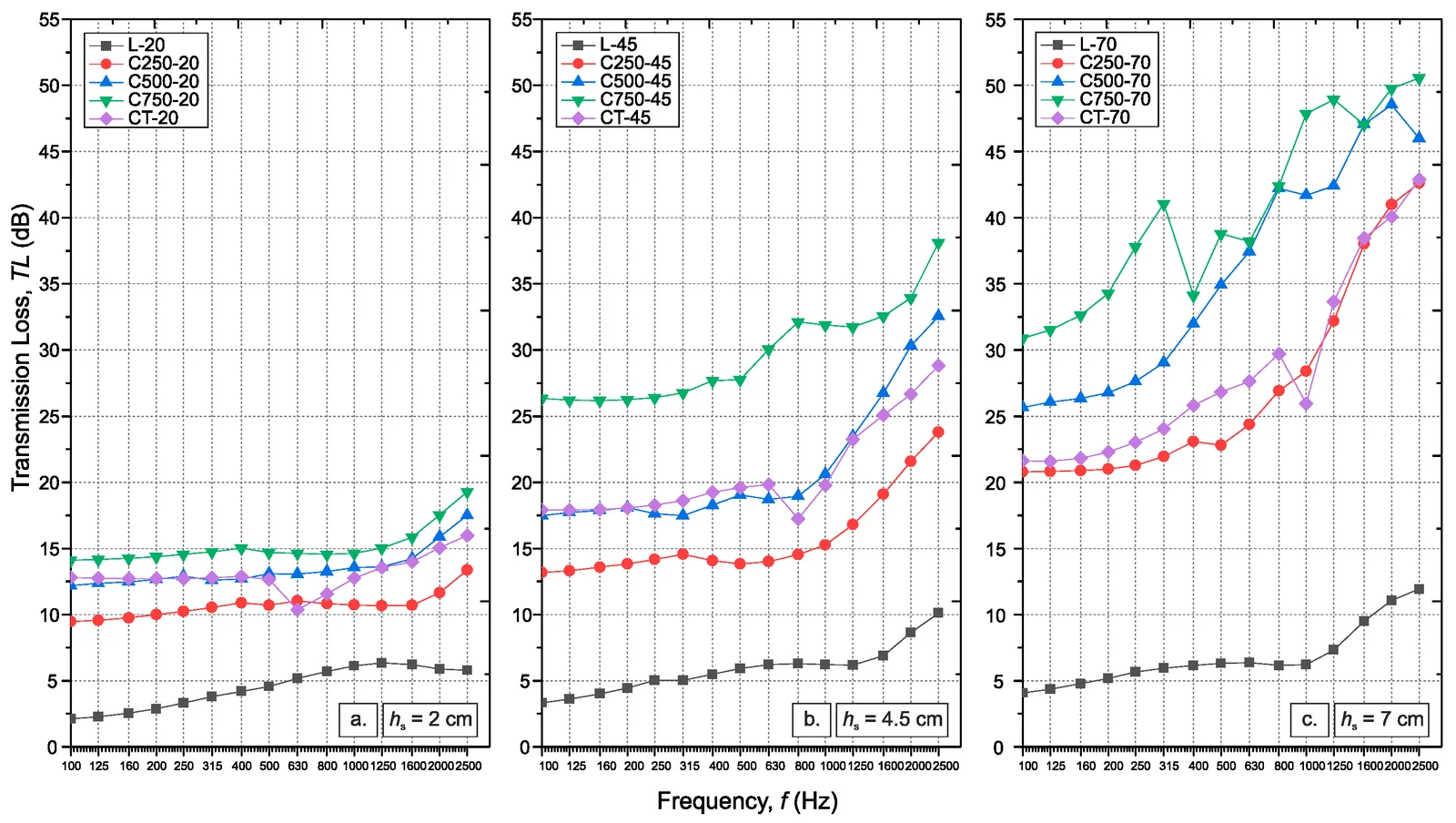
Frequency dependence of transmission loss, TL, for loose and compact textile specimens at different specimen thicknesses: (**a**) *h_s_* = 2 cm; (**b**) *h_s_* = 4.5 cm; and (**c**) *h_s_* = 7 cm.

Figure 5 shows the frequency dependence of transmission loss TL for the automotive textile recyclate over the range 100–2500 Hz for specimens L, C250, C500, C750, and CT at thicknesses of *h_s_* = 2, 4.5, and 7 cm. In all cases, specimen L exhibited the lowest TL values, indicating the lowest sound-insulation performance of the loose textile fraction without additional mechanical compression. As the compaction pressure increased, TL progressively increased, with the most pronounced effect observed at *h_s_* = 7 cm. At this thickness, specimens C500 and C750 achieved higher TL values than the compact textile specimens CT.

Measurement repeatability was evaluated using the standard deviation (SD) of the five technical repetitions in the individual one-third-octave bands. For specimens with a thickness of 20 mm, the SD did not exceed 0.12 dB. At a thickness of 45 mm, it did not exceed 0.25 dB, except for specimen C750-45, for which it reached 0.53 dB at 100 Hz. Higher maximum SD values were recorded for some configurations with a thickness of 70 mm, specifically 0.45 dB for C250-70, 1.53 dB for C500-70, 1.37 dB for C750-70, and 0.96 dB for CT-70. However, the median SD across the evaluated frequency bands did not exceed 0.41 dB for any configuration.

**Figure 6:**
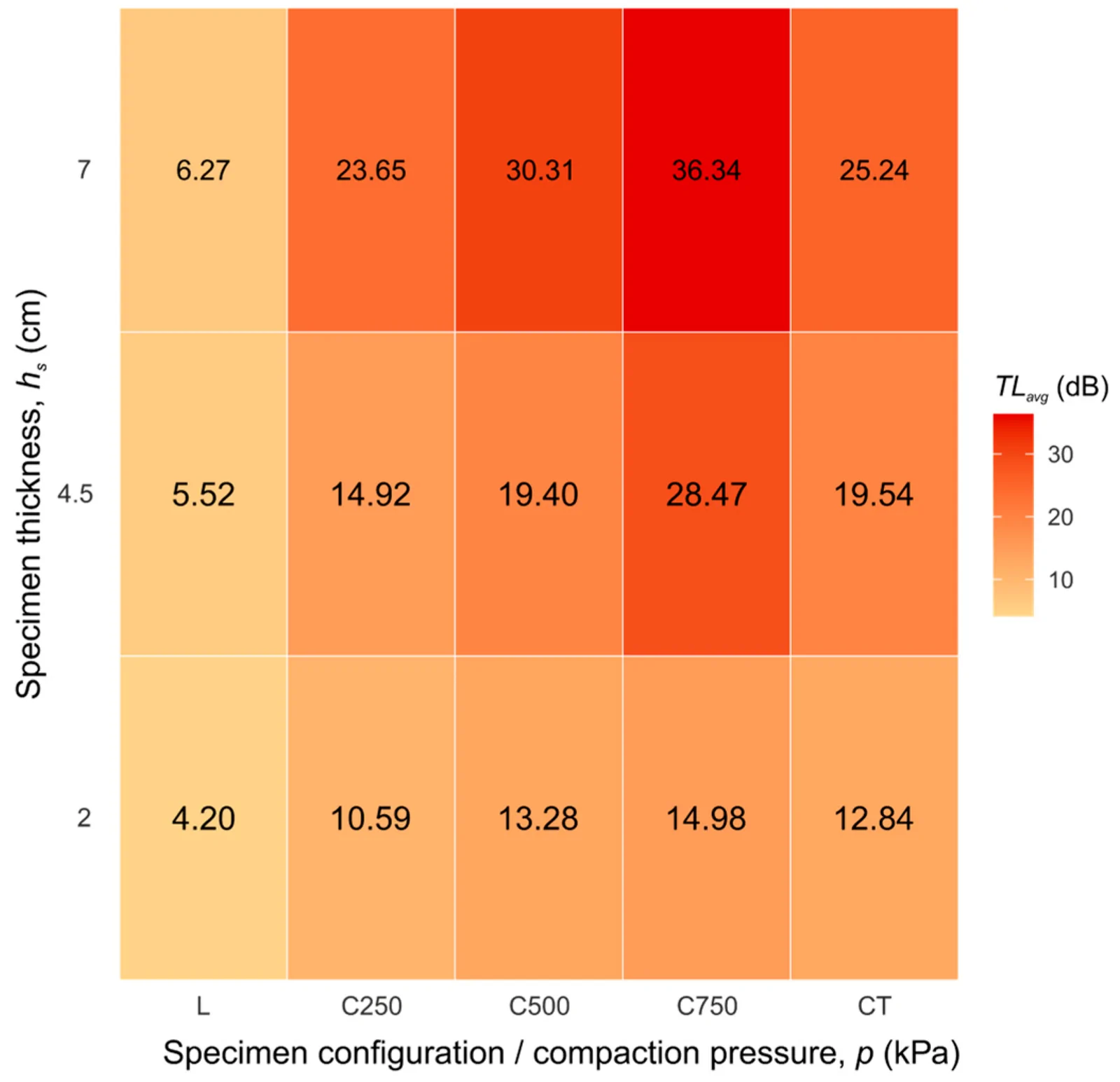
Average transmission loss, *TL_avg_* (dB), of automotive textile recyclate as a function of specimen configuration and specimen thickness, *h_s_*. L denotes the uncompacted loose textile fraction; C250, C500 and C750 denote loose textile fractions compacted at 250, 500 and 750 kPa, respectively; CT denotes compact textile specimens.

The average transmission-loss values $TL_{avg}$ (in dB) were calculated using Equation (5) and are presented in Figure 6. The results confirm a systematic increase in TL with increasing compaction pressure and specimen thickness, indicating a substantial improvement in the sound-insulation properties of specimens made from the loose textile fraction. The lowest values were obtained for specimen L (*p* = 0 kPa), whereas specimen C750 exhibited the highest values. At *p* = 500 kPa, the values for the loose-textile-fraction specimens approached those of CT, while at *p* = 750 kPa they exceeded the CT values at all thicknesses. The highest sound-insulation performance was obtained for the combination of C750 and *h_s_* = 7 cm.

The standard deviation of *TL_avg_* did not exceed 0.25 dB for any evaluated configuration.

Based on the average TL values, the following ranking of sound-insulation performance can be established for each thickness:

− for *h_s_* = 2 cm: L < C250 < CT ≈ C500 < C750,
− for *h_s_* = 4.5 cm: L < C250 < C500 ≈ CT < C750,
− for *h_s_* = 7 cm: L < C250 < CT < C500 < C750.

These results show that compact textile specimens (CT) provide better sound-insulation performance than the loose textile fraction (L) without additional mechanical compression. At higher compaction pressures, particularly *p* = 500 and 750 kPa, however, specimens made from the loose textile fraction achieve TL values comparable to or higher than those of CT. The transition to comparable performance occurs at approximately *p* = 500 kPa, whereas at *p* = 750 kPa, the loose-textile-fraction specimens achieve higher TL values than CT at all evaluated thicknesses.

The effect of thickness alone was relatively limited for specimen L. Increasing the thickness from *h_s_* = 2 cm to *h_s_* = 7 cm increased $TL_{avg}$ only from 4.20 to 6.27 dB. In contrast, combining greater thickness with higher pressure produced a substantially larger improvement. For specimen C750, $TL_{avg}$ increased from 14.98 dB at *h_s_* = 2 cm to 36.34 dB at *h_s_* = 7 cm. This confirms that the sound-insulation performance of the loose textile fraction is strongly affected by the interaction between specimen thickness and compaction pressure.

Overall, higher transmission loss TL corresponds to lower acoustic-energy transmission and better sound-insulation performance. The best results were obtained for specimen C750 with a thickness of *h_s_* = 7 cm, which exhibited the highest average TL value. The combination of greater thickness and higher nominal pressure therefore represents the most effective configuration for improving the sound-insulation properties of automotive textile recyclate.

#### Relationship Between Transmission Loss and Areal Mass

**Figure 7:**
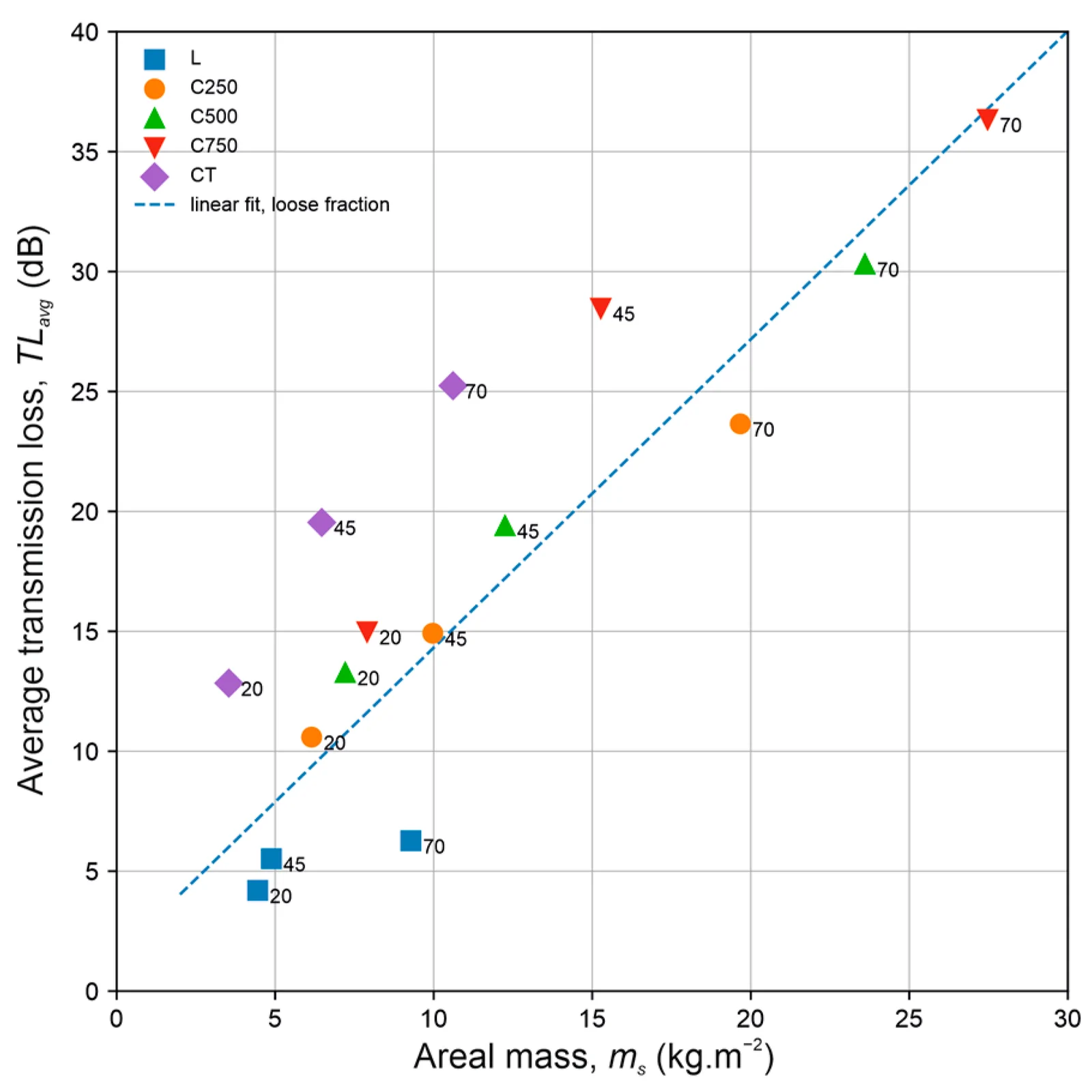
Average transmission loss, *TL_avg_*, as a function of areal mass, *m_s_*, for loose textile fraction specimens (L, C250, C500, C750) and compact textile specimens (CT). Numbers next to the symbols denote specimen thickness in mm; the dashed line represents the linear fit for the loose textile fraction.

Because the compacted specimens were prepared at defined thicknesses, higher compaction pressure was necessarily associated with a greater amount of material in the test cassette and therefore with higher bulk density and areal mass (Table 3). To partially separate the relationship of compaction from the effect of the amount of material, *TL_avg_* was evaluated in relation to the areal mass *m_s_* of the individual specimens (Figure 7).

For the loose textile fraction specimens, areal mass alone explained a substantial proportion of the variability in *TL_avg_* (linear regression, *n* = 12, *R*^2^ = 0.87; regression coefficient for areal mass = 1.29 dB per kg·m^−2^). After compaction pressure was included in the model, the explained variability increased to *R*^2^ = 0.97, and compaction pressure remained significantly associated with *TL_avg_* (*p* < 0.001); after accounting for areal mass, the model estimated an increase in *TL_avg_* of approximately 1.3 dB per 100 kPa. Thus, compaction pressure remained associated with TL even after accounting for areal mass. However, because of the correlation between *p* and *m_s_* (*r* = 0.53) and the limited number of experimental configurations, the two effects cannot be completely separated.

The compact CT specimens lay above the trend observed for the loose textile fraction. At comparable areal mass, they achieved higher values of *TL_avg_*. The compacted loose-textile-fraction specimens that, at the same thickness, achieved or exceeded the TL values of the compact CT specimens had an areal mass corresponding to approximately 1.9 to 2.6 times the areal mass of the respective CT specimens. In addition, the CT specimens differ from the loose textile fraction not only in bulk density and areal mass, but also in their coherent bonded structure, the presence of a PUR binder, and the preparation method.

### 3.3. Regression Models and Determination of Variable Importance

#### 3.3.1. Regression Models

To quantitatively assess the effects of the investigated variables on transmission loss TL, linear regression models were developed for specimens made from the loose textile fraction (L, C250, C500, and C750) and for compact textile specimens (CT). The regression analysis was intended to determine which variables and interaction effects most strongly influenced TL over the frequency range 100–2500 Hz. For specimens made from the loose textile fraction, the input variables were frequency *f*, compaction pressure *p*, and specimen thickness *h_s_*. Compaction pressure was not included as an input variable for the compact textile specimens because the parameter *p* applies only to specimens made from the loose textile fraction. The CT model therefore included only frequency *f* and specimen thickness *h_s_*.

The regression models developed in this study are empirical, and their validity is limited to the investigated automotive textile recyclate and the experimental range of frequencies, thicknesses, and compaction states used. Their application outside this range or to other materials requires further experimental verification.

For specimens made from the loose textile fraction, the following general relationship was considered:

(8) $$TL=g\left(f,p,h_{s}\right)$$

where TL is the transmission loss, *g* is a general function describing the dependence of sound transmission loss on the input variables, *f* is frequency, *p* is compaction pressure, and *h_s_* is the specimen thickness used in the statistical evaluation. For compact textile specimens, the model was defined without compaction pressure:

(9) $$TL=g\left(f,h_{s}\right).$$

First, a basic linear model without interaction terms, designated Model I, was developed for specimens made from the loose textile fraction:

(10) $$TL=\beta_{0}+\beta_{1}\cdot f+\beta_{2}\cdot p+\beta_{3}\cdot h_{s}+\varepsilon$$

Model I served as the baseline model for assessing the individual effects of frequency, compaction pressure, and specimen thickness. An extended model including interaction terms, designated Model II, was then developed for specimens made from the loose textile fraction:

(11) $$TL=\beta_{0}+\beta_{1}\cdot f+\beta_{2}\cdot p+\beta_{3}\cdot h_{s}+\beta_{4}\cdot \left(f\times p\right)+\beta_{5}\cdot \left(f\times h_{s}\right)+\beta_{6}\cdot \left(p\times h_{s}\right)+\varepsilon$$

Model II captures the combined effects of the investigated variables, particularly the interactions between frequency and compaction pressure, frequency and specimen thickness, and compaction pressure and specimen thickness.

A separate model, designated Model III, was developed for the compact textile specimens (CT):

(12) $$TL=\beta_{0}+\beta_{1}\cdot f+\beta_{2}{\cdot h}_{s}+\beta_{3}\cdot \left(f\times h_{s}\right)+\varepsilon$$

Model III includes frequency, specimen thickness, and the effect of their interaction, whereas compaction pressure is not considered an input variable. In all models, $\beta_{0}$ is the intercept, $\beta_{i}$ are the regression coefficients, and ε is the random model error.

Point estimates of the regression-model parameters, standardized regression coefficients *β*, standard errors of the estimates, and statistical-significance values (*p*-value) are presented in Table 5. Statistical significance was assessed at the significance level α=0.05. In the regression modeling, *h_s_* values were entered in meters to maintain consistency with the International System of Units (SI) and to express the regression coefficients as the change in transmission loss per unit change in specimen thickness.

All *p*-values reported in Table 5 were calculated using standard errors obtained by ordinary least squares (OLS) estimation. Multicollinearity among the explanatory variables was evaluated using the variance inflation factor (VIF). In Model I, all predictors had VIF values of approximately 1.0, indicating no multicollinearity. In Models II and III, VIF values ranged from 2.21 to 7.78. The elevated values were expected because interaction terms were included in the regression models; however, all values remained below the threshold of 10, indicating no severe multicollinearity. Because model diagnostics indicated a violation of the homoscedasticity assumption, robust standard errors were calculated as a sensitivity check. The robust analysis confirmed the statistical significance of most regression coefficients. The *f* × *p* interaction in Model II was statistically significant under ordinary least squares estimation (*p*-value = 0.003). After robust standard errors were taken into account, however, it showed borderline statistical significance (*p*-value = 0.051), while the principal conclusions of the regression models remained stable.

The basic model for specimens made from the loose textile fraction, designated Model I, explained a high proportion of the variability ($R^{2}$ = 0.831; *p*-value < 2 × 10^−16^). All variables considered—frequency, compaction pressure, and specimen thickness—were statistically significant in this model (*p*-value < 0.001). This result confirms that each variable independently contributes to changes in transmission loss. However, this model does not account for the effects of interactions between the variables. An extended model including interaction terms, designated Model II, was therefore developed for specimens made from the loose textile fraction. This model had substantially greater explanatory power ($R^{2}$ = 0.933; *p*-value < 2 × 10^−16^), confirming the importance of the interactions. After the interaction terms were included, the individual effects of compaction pressure (*p*) and thickness (*h_s_*) were no longer statistically significant (*p*-value > 0.05). In contrast, the interaction terms were statistically significant, indicating that the effects of pressure and thickness cannot be interpreted independently. The most important, combined effect was that of pressure and thickness (*p* × *h_s_*). The interaction between frequency and thickness (*f* × *h_s_*) was also significant, as was, to a lesser extent, the interaction between frequency and pressure (*f* × *p*). A separate regression model (Model III) was developed for the compact textile specimens (CT) and it showed very high explanatory power ($R^{2}$ = 0.976; *p*-value < 2 × 10^−16^). All variables considered were statistically significant (*p*-value < 0.05). The interaction between frequency and specimen thickness (*f* × *h_s_*) played the dominant role, indicating a pronounced frequency–geometry dependence of transmission loss in the compact material form.

The dominant interaction term differed between the two material forms. For specimens made from the loose textile fraction, the combined effect of compaction pressure and specimen thickness had the highest Std. *β* value, whereas for compact textile specimens, the interaction between frequency and specimen thickness had the highest Std. *β* value.

The out-of-specimen predictive performance of Models I and II was evaluated using leave-one-specimen-out cross-validation (LOSO-CV) at the physical-specimen level. The cross-validation results are summarized in Table 6. Model III was not subjected to specimen-level cross-validation because only three physical CT specimens were available.

**Figure 8:**
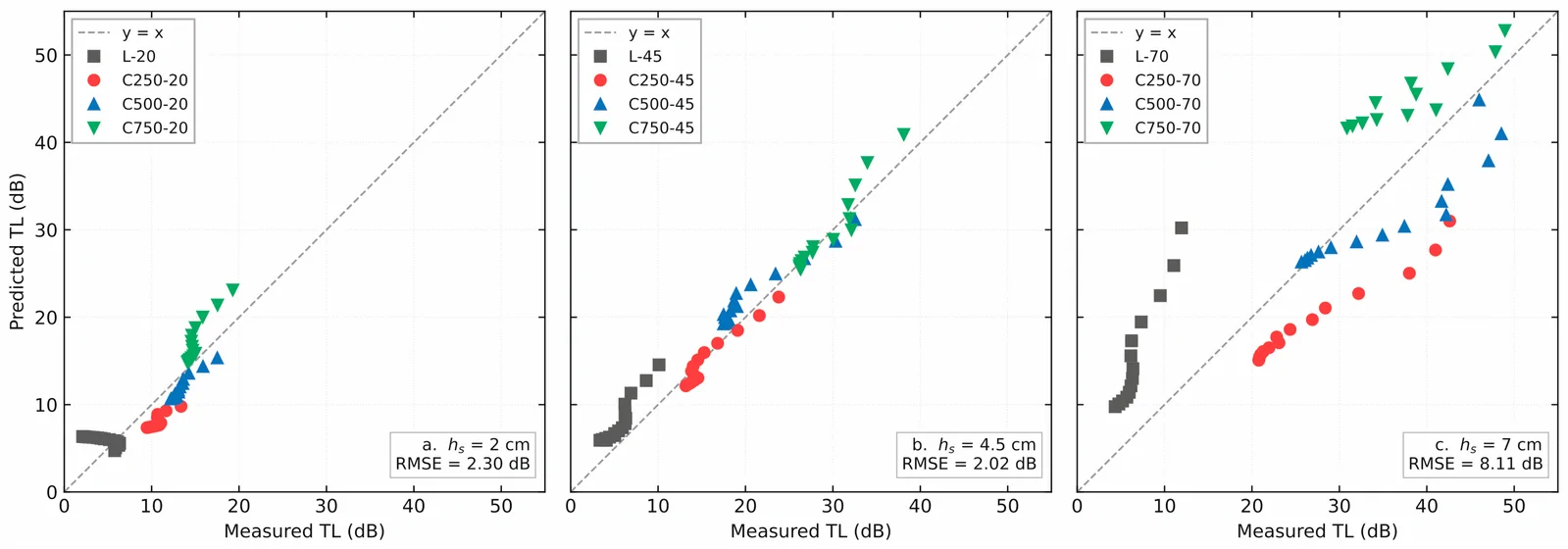
Leave-one-specimen-out cross-validation of Model II: comparison of measured and predicted TL values for specimen thicknesses of (**a**) *h_s_* = 2 cm, (**b**) *h_s_* = 4.5 cm, and (**c**) *h_s_* = 7 cm. The dashed line represents perfect agreement (*y* = *x*). RMSE values are shown separately for each thickness.

The cross-validated predictive performance was lower than the fit obtained using all available specimens, as expected for out-of-specimen prediction. Model II showed better predictive performance than Model I, with R^2^_CV_ = 0.836, RMSE = 4.97 dB, and MAE = 3.57 dB, compared with R^2^_CV_ = 0.688, RMSE = 6.85 dB, and MAE = 5.03 dB for Model I. The cross-validated predictions of Model II are shown in Figure 8. These results indicate that inclusion of the interaction terms improved the prediction of TL for specimens excluded from model fitting. Model III was not evaluated by specimen-level cross-validation because only three physical CT specimens were available.

For Model II, the cross-validated predictions were generally in good agreement with the measured TL values, although the prediction accuracy varied with specimen thickness (Figure 8). Prediction errors depended markedly on specimen thickness: RMSE values were 2.30, 2.02, and 8.11 dB and MAE values were 2.02, 1.66, and 7.10 dB for thicknesses of 20, 45, and 70 mm, respectively. The highest prediction errors therefore occurred at the greatest investigated thickness, *h_s_* = 70 mm. Under leave-one-specimen-out validation, after one 70-mm specimen was excluded, only three specimens of this thickness remained in the training dataset. Configurations L-70 and C750-70 additionally represent corner points of the experimental design because they combine the greatest thickness with the lowest and highest compaction pressure, respectively. As shown in Figure 8c, Model II overpredicted TL for L-70 and C750-70, whereas TL for C250-70 was predominantly underpredicted. The pattern of prediction errors at *h_s_* = 70 mm therefore varied with compaction pressure, suggesting that the linear interaction terms of Model II do not fully capture the pressure dependence of TL at the greatest investigated thickness. At thicknesses of 20 and 45 mm, the cross-validated predictions were in substantially better agreement with the measured values, consistent with the markedly lower RMSE values in Figure 8a,b. The cross-validation therefore indicates that the models adequately describe TL within the investigated experimental range, while predictive accuracy decreases at the greatest investigated thickness, particularly for the corner configurations of the experimental design. Because the cross-validation was based on twelve specimens from a single material batch, the predictive performance of the models should be further verified using independently prepared specimens and additional material batches. Accordingly, LOSO-CV should be regarded as an internal assessment of out-of-specimen predictive performance within the investigated material batch rather than as external validation.

#### 3.3.2. Variable Importance Based on Standardized Regression Coefficients

The importance of the individual variables was evaluated using standardized regression coefficients (Std. *β*). These coefficients enable the relative strength of the effects of individual variables and interaction terms within a regression model to be compared. A positive *β* value indicates that an increase in the variable or interaction term is associated with an increase in TL, whereas a negative *β* value indicates the opposite direction of effect. An overview of the interpretation of the individual variables and interaction terms is provided in Table 7.

In the basic model for specimens made from the loose textile fraction (Model I), all main variables had a positive effect on transmission loss. Compaction pressure had the strongest individual effect (Std. *β* ≈ 0.667), confirming its pronounced individual effect in the model without interactions. Specimen thickness also had a substantial positive effect (Std. *β* ≈ 0.552), whereas frequency had only a modest positive effect (Std. *β* ≈ 0.246). After the interaction terms were included in Model II, the main effects of compaction pressure and specimen thickness were no longer statistically significant. The interaction term *p* × *h_s_* had the highest standardized coefficient, indicating that the relationship between compaction pressure and TL depends on specimen thickness. Compaction pressure and specimen thickness therefore represent interrelated experimental factors in this model, and their effects on TL cannot be interpreted separately. The value Std. *β* ≈ 0.764 confirms that the interaction term *p* × *h_s_* had the strongest standardized regression effect among the terms in Model II and indicates that the effect of compaction changes with specimen thickness. The interaction between frequency and specimen thickness (*f* × *h_s_*) also had a substantial positive effect. Its Std. *β* value of approximately 0.406 confirms its important contribution to changes in TL. The individual effect of frequency was slightly negative in this model (Std. *β* ≈ −0.141), suggesting that its influence is expressed primarily through interactions with the other variables. For compact textile specimens (CT), the interaction between frequency and specimen thickness (*f* × *h_s_*) had the largest absolute standardized regression coefficient in Model III. The Std. *β* value of approximately 0.806 indicates a strong standardized effect and highlights the key role of the frequency–geometry dependence in the compact material form. Specimen thickness also had a substantial positive effect on *TL* (Std. *β* ≈ 0.479), whereas frequency had a modest negative individual effect (Std. *β* ≈ −0.198). This result shows that, for compact textile specimens, the effect of frequency is expressed primarily in combination with specimen thickness.

From a physical perspective, the individual variables of the regression models can be interpreted in relation to the experimentally observed sound transmission. Frequency (*f*) expresses the frequency dependence of TL, whereas specimen thickness (*h_s_*) characterizes the path length over which acoustic energy propagates through the material layer. Compaction pressure (*p*) represents the experimentally controlled degree of compression of the loose textile fraction; increasing p was associated with an increase in bulk density and can be physically related to changes in fiber arrangement, contacts among textile particles, and the volume of air voids. The interaction *f × p* expresses that the frequency dependence of TL may change with the degree of compaction. The interaction *f × h_s_* interaction expresses that the effect of frequency depends on material-layer thickness, which is consistent with frequency-dependent sound propagation through a layer of finite thickness. The interaction *p × h_s_* interaction indicates that the effect of compaction on TL changes with specimen thickness. This relationship can be physically associated with the combined effect of changes in material arrangement and bulk density during compaction and the increasing thickness of the layer through which acoustic energy is transmitted. Because parameters characterizing the internal material structure were not directly measured in this study, these relationships should be regarded as a physically justified interpretation of the statistical results rather than as experimental confirmation of specific sound-propagation mechanisms.

Comparison of the models indicates that the relative importance of the investigated variables and their interactions differ between the evaluated forms of automotive textile recyclate. For specimens made from the loose textile fraction, the combined effect of compaction pressure and specimen thickness is particularly important, supporting the use of Model II to interpret interaction effects. For compact textile specimens (CT), the interaction between frequency and specimen thickness predominates; Model III is therefore the key model for this material form. Model I primarily provides information on the individual effects of the variables because it does not include their interactions. These results show the relative strength of the regression effects, rather than the shares of variability explained by the model that are attributable to individual predictors, which are evaluated separately by dominance analysis in Section 3.3.4. From the standpoint of TL optimization, the individual variables therefore cannot be evaluated in isolation; their combined effects must be considered.

#### 3.3.3. Response Surface Analysis

Response surfaces were generated from the regression models to visualize the combined effects of the investigated variables and to facilitate interpretation of the interaction effects identified in the preceding section. For specimens made from the loose textile fraction, the response surfaces were based on Model II, which includes both main effects and interaction terms. For compact textile specimens (CT), the response surface was based on Model III.

**Figure 9:**
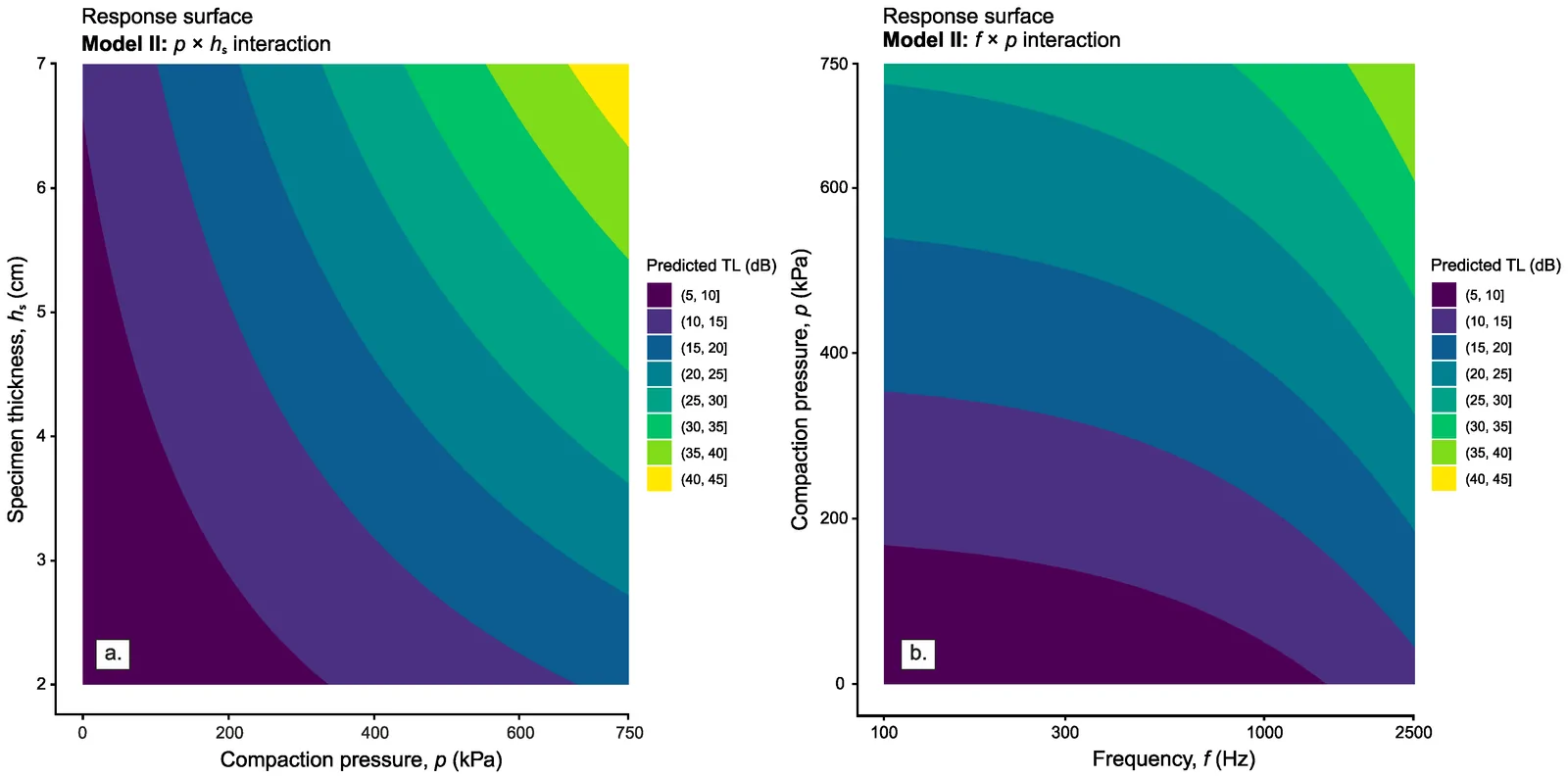
Response surface plots predicted by Model II for loose textile fraction specimens, showing the (**a**) effect of compaction pressure and specimen thickness on TL; (**b**) effect of frequency and compaction pressure on TL.

Figure 9 shows the Model II response surfaces for specimens made from the loose textile fraction. The most pronounced effect was observed for the interaction between compaction pressure (*p*) and specimen thickness (*h_s_*); see Figure 8a. Higher predicted TL values were obtained at greater compaction pressures and specimen thicknesses, with the maximum occurring when both variables were at their highest levels. The curvature of the contour lines indicates a pronounced interaction between *p* and *h_s_*, demonstrating that the effect of compaction pressure changes with specimen thickness. This result is consistent with the standardized regression coefficients, according to which the *p* × *h_s_* interaction term had the highest Std. *β* value in Model II. The response surface in Figure 8b shows the combined effect of frequency and compaction pressure on transmission loss. At low compaction pressures, the predicted TL values were lower, whereas they increased with increasing pressure. The effect of frequency was most evident at higher compaction pressures, suggesting that compaction may increase the frequency sensitivity of specimens made from the loose textile fraction. However, this effect was weaker than the interactions involving specimen thickness. After robust standard errors were considered, the *f* × *p* interaction was only marginally statistically significant; it was therefore interpreted cautiously and was not considered to be a dominant factor affecting TL.

**Figure 10:**
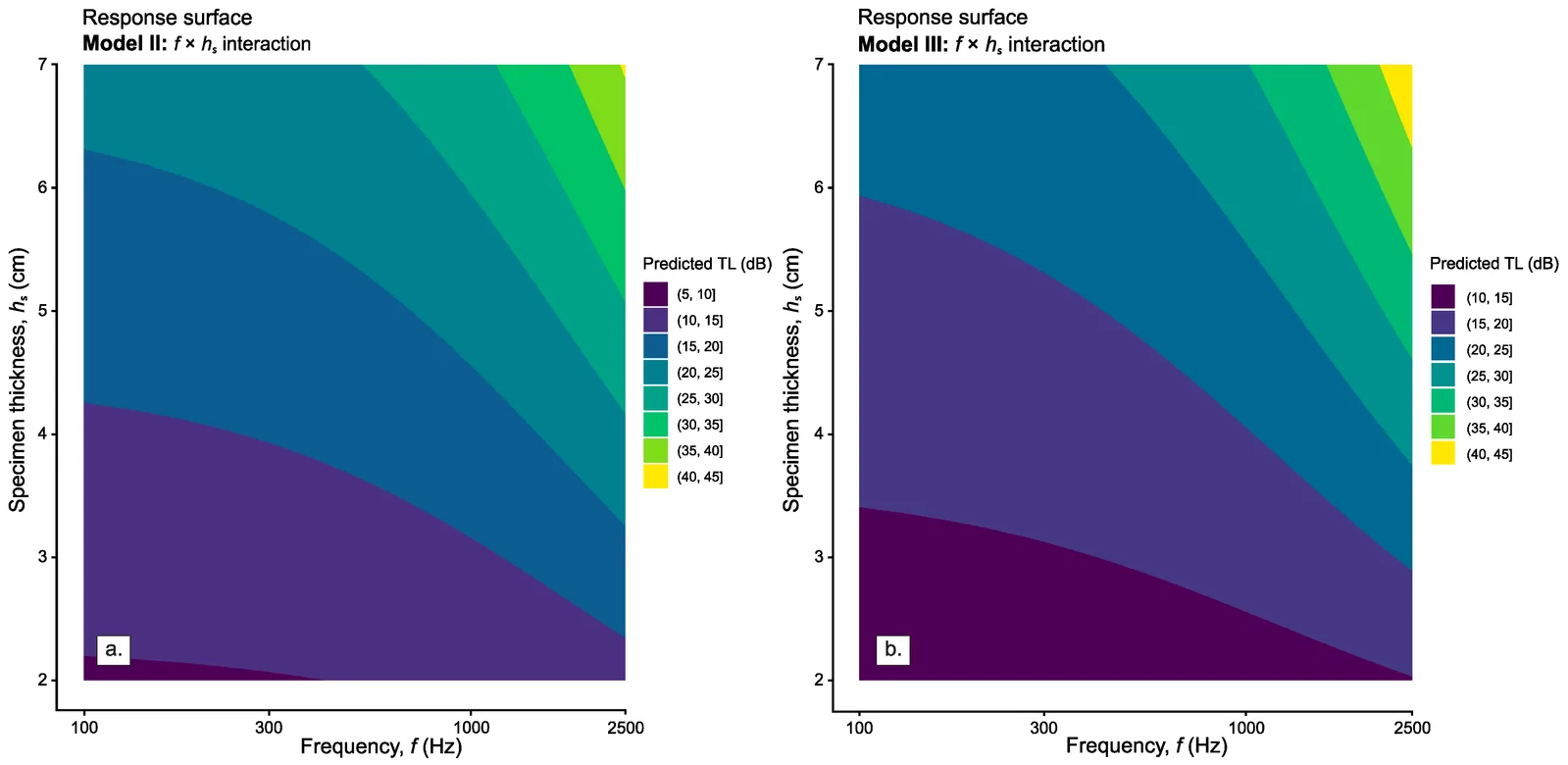
Response surface plots showing the predicted effect of frequency and specimen thickness on transmission loss, TL: (**a**) loose textile fraction specimens, Model II; (**b**) compact textile specimens, Model III.

Figure 10 enables the frequency–geometry dependence of the predicted TL values to be compared for specimens made from the loose textile fraction and for compact textile specimens, that is, the combined effect of frequency and specimen thickness. In both cases, TL increases toward higher levels of both variables, with the maximum occurring when they are combined. However, the intensity of this trend differs between the evaluated material forms. For compact textile specimens (Figure 10b), the frequency–geometry dependence is more pronounced, and the *f* × *h_s_* interaction term represents the dominant interaction affecting TL in Model III. For specimens made from the loose textile fraction (Figure 10a), this interaction is also present, but its importance in Model II is lower than that of the interaction between compaction pressure and specimen thickness. Thus, for the loose textile fraction, the relationship between TL and the investigated variables is dominated by the interaction between compaction pressure and specimen thickness, whereas the interaction between frequency and specimen thickness predominates for compact textile specimens (CT).

The response surfaces visually support the different interaction patterns identified for the two material forms: the *p* × *h_s_* interaction predominates for the loose textile fraction, whereas the *f* × *h_s_* interaction predominates for compact textile specimens (CT). The response-surface analysis therefore supports the conclusions of the regression evaluation.

#### 3.3.4. Variable Importance Based on Dominance Analysis

Dominance analysis was used to quantify the relative contributions of the individual variables and interaction terms to the explained variability in transmission loss TL. Unlike the standardized regression coefficients, Std. *β*, which indicate the direction and relative strength of the effect of each model term, dominance analysis evaluates the shares of variability explained by the model that are attributable to each predictor, expressed by *R*^2^. This analysis therefore complements the interpretation based on the regression coefficients and response surfaces. Dominance analysis was applied to the extended regression models, namely Model II for specimens made from the loose textile fraction and Model III for compact textile specimens (CT). The explained variability was *R*^2^ = 0.933 for Model II, whereas Model III achieved *R*^2^ = 0.976. Dominance analysis assigns only the portion of TL variability captured by the regression model; the unexplained portion is therefore not included in this analysis.

**Figure 11:**
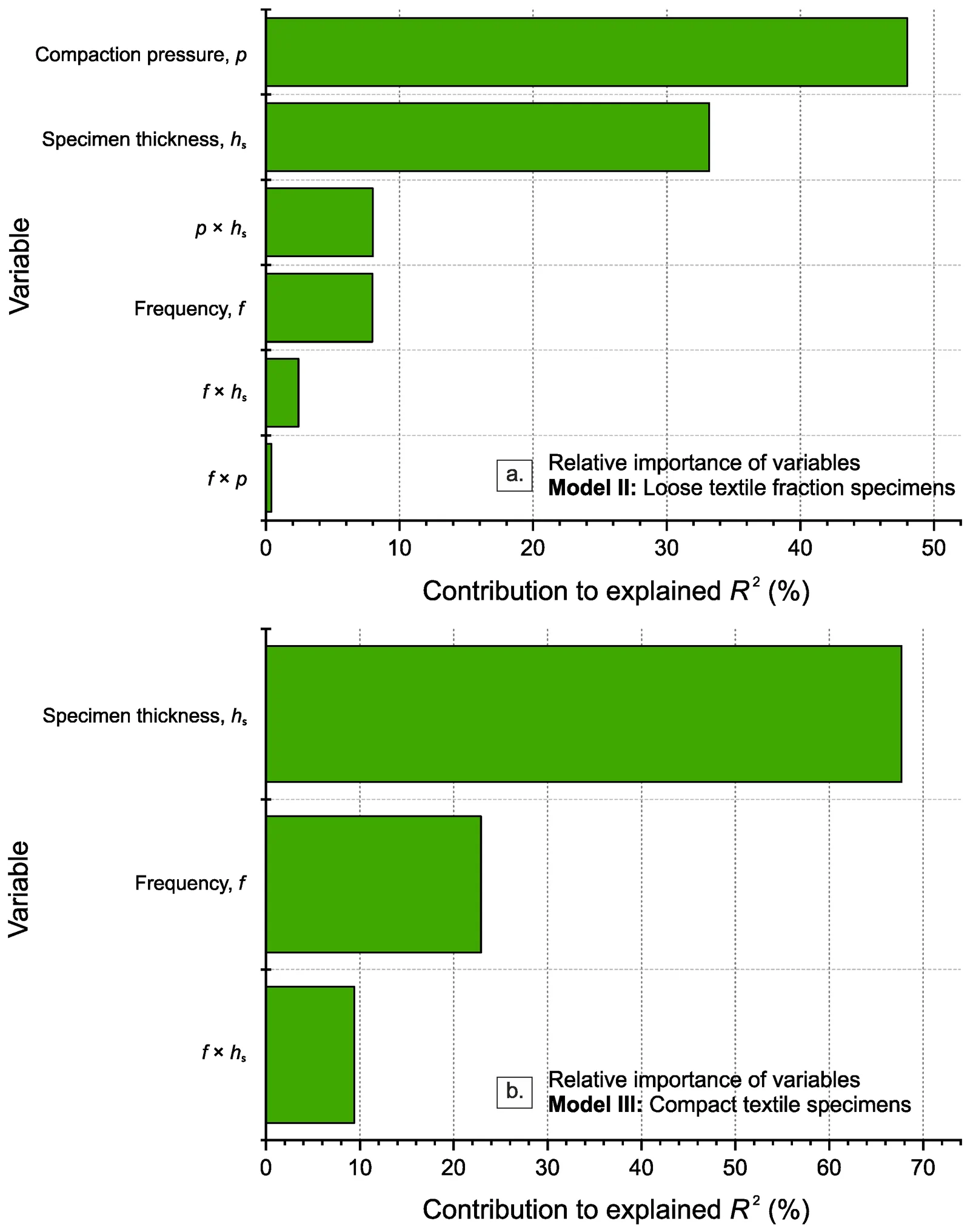
Dominance analysis of variables contributing to the explained variability of transmission loss, TL: (**a**) loose textile fraction specimens, Model II; (**b**) compact textile specimens, Model III.

Figure 11a shows the distribution of explained TL variability among the individual variables and interaction terms in Model II for specimens made from the loose textile fraction. Compaction pressure *p* made the largest contribution, accounting for approximately 48% of the variability explained by the model, followed by specimen thickness *h_s_*, which contributed approximately 33%. Frequency *f* made a smaller individual contribution of approximately 8%. Among the interaction terms, *p* × *h_s_* made the largest contribution, accounting for approximately 8% of the explained variability. The *f* × *h_s_* interaction made only a minor contribution (≈2.4%), and the contribution of the *f* × *p* interaction was almost negligible. For compact textile specimens (CT), dominance analysis showed a different ranking of variable importance; see Figure 11b. Specimen thickness made the largest contribution to the explained variability (*h_s_* ≈ 65–70%), confirming its key role as the principal source of TL variability in Model III. Frequency was a secondary factor (*f* ≈ 23%), whereas the interaction between frequency and specimen thickness (*f* × *h_s_* ≈ 8–10%) made a smaller contribution to the explained variability. This result shows that, for compact textile specimens (CT), specimen thickness determines the overall magnitude of changes in TL, whereas the *f* × *h_s_* interaction describes the frequency-dependent nature of the thickness effect.

The dominance-analysis results complement the interpretation based on standardized regression coefficients by distinguishing the principal sources of TL variability from the terms that shape the form of the relationship. A high Std. *β* value for an interaction term therefore does not necessarily imply the largest share of variability explained by the model. The main variables primarily determine the overall magnitude of changes in TL, whereas the interaction terms describe how the effect of one variable changes as a function of another.

From a practical perspective, the results show that TL optimization cannot be based solely on independently setting the individual parameters. For specimens made from the loose textile fraction, compaction pressure and specimen thickness must be considered simultaneously because these variables are the principal sources of explained TL variability and also form the dominant *p* × *h_s_* interaction (the highest Std. *β* value in Model II). For compact textile specimens (CT), specimen thickness is the principal design parameter, and its effect depends on frequency.

### 3.4. Transmission Coefficient and Comparison Using Relative Transmission Ratio

After transmission loss TL had been evaluated, the results were further analyzed using the transmission coefficient $\tau_{f}$, calculated from *TL_f_* values in accordance with Equation (3), and the relative transmission ratio *RTR_f_*, defined by Equation (6). The transmission coefficient $\tau_{f}$ enables the results to be interpreted in terms of the fraction of acoustic energy transmitted through the test specimen, whereas *RTR_f_* enables direct comparison of transmission behavior among selected configurations at the same frequency.

#### 3.4.1. Transmission Coefficient

The transmission coefficient $\tau_{f}$ was calculated from the measured *TL_f_* values to express acoustic-energy transmission on a linear scale. Because *TL_f_* is defined on a logarithmic scale, conversion to $\tau_{f}$ enables the fraction of acoustic energy transmitted through the individual specimens at a given frequency to be compared directly. Higher $\tau_{f}$ values correspond to greater acoustic-energy transmission through the material, whereas lower $\tau_{f}$ values indicate higher transmission loss. For clearer interpretation, the transmission-coefficient values were expressed as percentages in the graphs.

**Figure 12:**
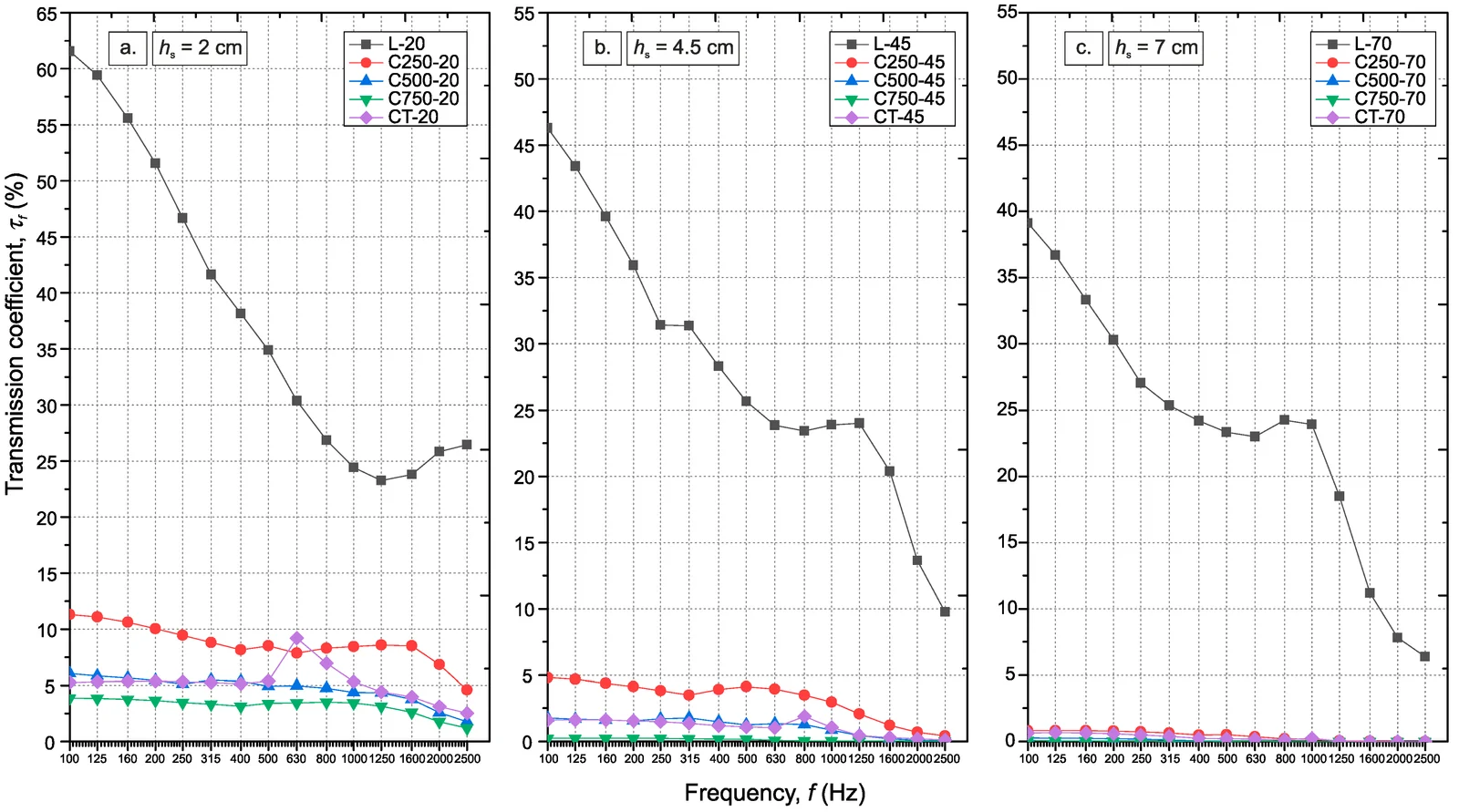
Transmission coefficient, τf, for loose and compact textile specimens at different specimen thicknesses: (**a**) *h_s_* = 2 cm; (**b**) *h_s_* = 4.5 cm; and (**c**) *h_s_* = 7 cm.

Figure 12 shows the frequency dependence of the transmission coefficient $\tau_{f}$ for specimens made from the loose textile fraction in the uncompacted state L and in the compacted states C250, C500, and C750, together with the compact textile specimens CT. The effects of compaction pressure, specimen thickness, and material form on acoustic-energy transmission are evaluated over the frequency range 100–2500 Hz. The individual plots represent specimen thicknesses of *h_s_* = 2.0, 4.5, and 7.0 cm.

At all evaluated thicknesses, the $\tau_{f}$ values decreased substantially across the entire frequency range as compaction pressure increased. The highest $\tau_{f}$ values were recorded for the uncompacted specimens L (*p* = 0 kPa), indicating that the uncompacted loose textile fraction transmitted the largest fraction of the incident acoustic energy. In contrast, the compacted specimens, particularly C500 and C750, exhibited substantially lower $\tau_{f}$ values, corresponding to higher transmission loss and lower acoustic-energy transmission through the material. For most configurations, $\tau_{f}$ decreased with increasing frequency, particularly for uncompacted or less-compacted specimens. This trend suggests that the ability of the material to limit acoustic-energy transmission increases not only with the degree of compaction but also as a function of frequency. The effect of compaction was most pronounced at greater thicknesses, where the combination of higher compaction pressure and greater specimen thickness produced the lowest $\tau_{f}$ values.

**Figure 13:**
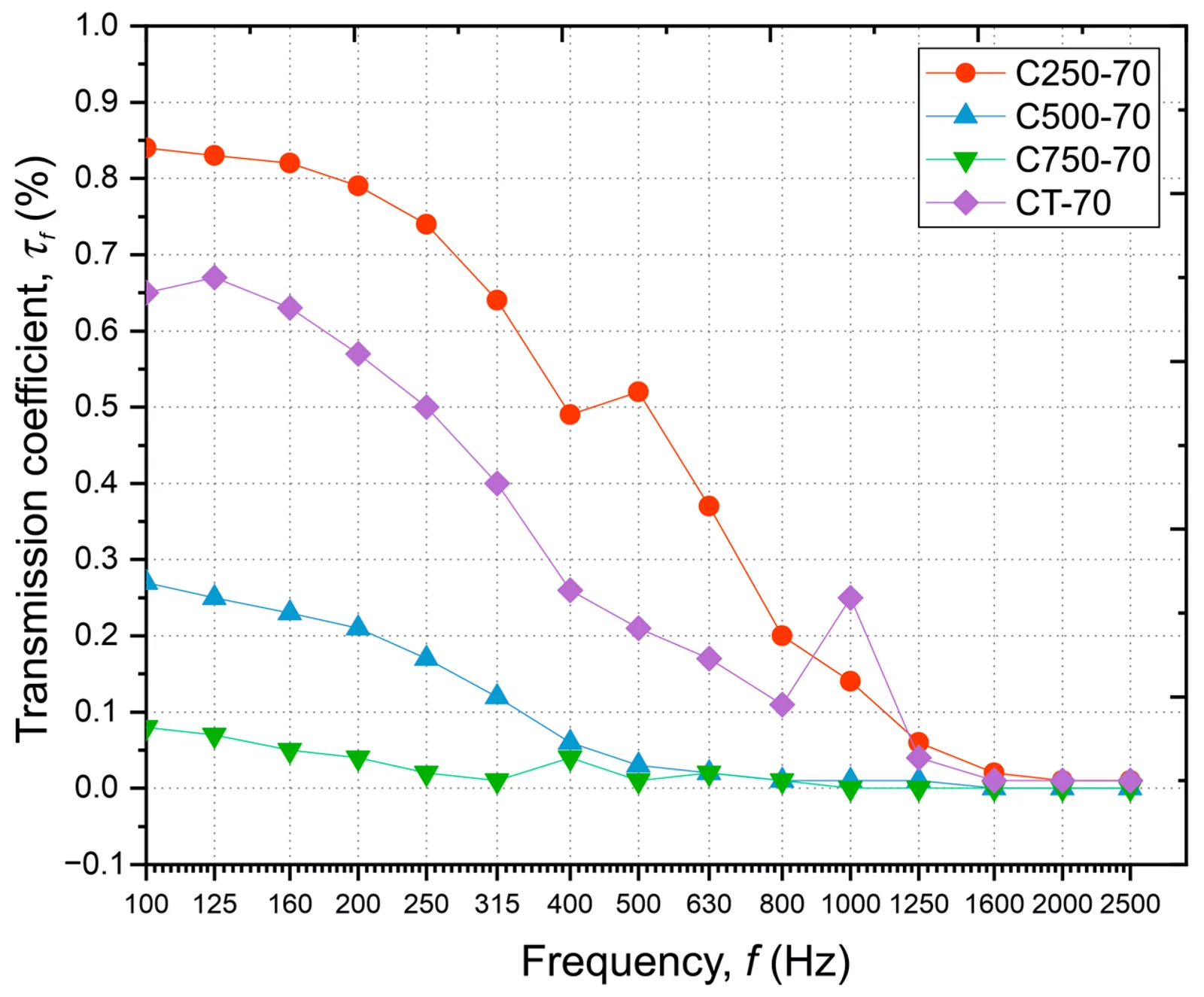
Transmission coefficient, *τ_f_*, for 7 cm specimens from compacted loose textile fractions and compact textile specimens.

At a thickness of *h_s_* = 7 cm and a compaction pressure of *p* = 750 kPa, the transmission coefficient $\tau_{f}$ reached its lowest values, with the percentage of transmitted acoustic energy approaching hundredths of a percent. This indicates very low acoustic-energy transmission and corresponds to very high transmission-loss values. Therefore, to improve readability and resolution, a separate graph was created for *h_s_* = 7 cm (see Figure 13), excluding the values for the uncompacted specimen L (*p* = 0 kPa).

**Figure 14:**
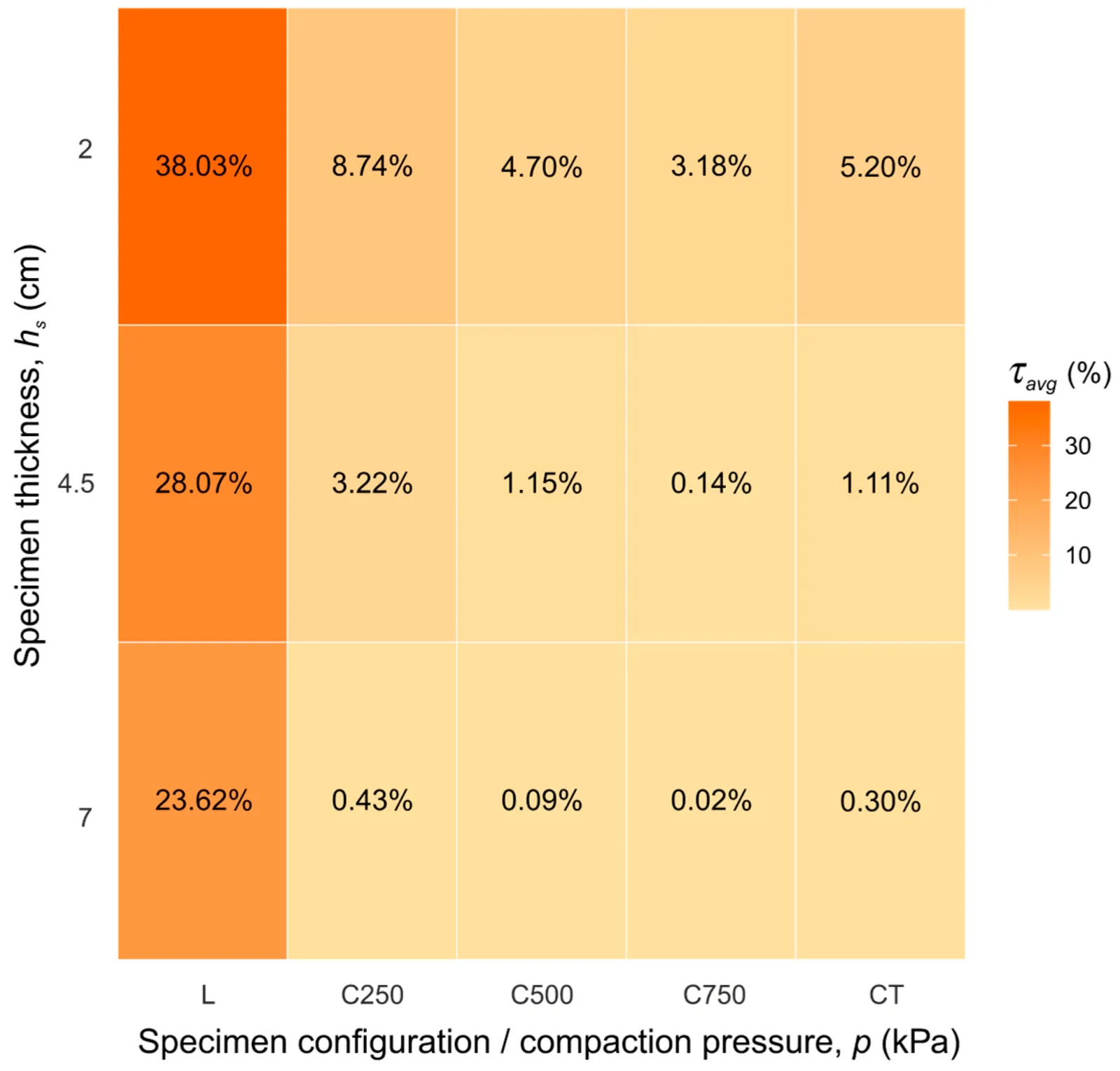
Average transmission coefficient, *τ_avg_* (%), of automotive textile recyclate as a function of specimen configuration and specimen thickness. L denotes the uncompacted loose textile fraction; C250, C500 and C750 denote loose textile fractions compacted at 250, 500 and 750 kPa, respectively; CT denotes compact textile specimens.

The average transmission-coefficient values $\tau_{avg}$, determined in accordance with Equation (4), are shown in Figure 14 for the individual specimen thicknesses and compaction-pressure levels, together with the values for compact textile specimens CT. Increasing compaction pressure caused a substantial decrease in $\tau_{avg}$, indicating a reduction in the average fraction of acoustic energy transmitted through the test specimen and, consequently, an increase in the acoustic attenuation provided by the material.

The highest $\tau_{avg}$ values were recorded for the uncompacted specimens L (*p* = 0 kPa), particularly at the smaller thicknesses, where the fraction of transmitted energy was greatest. Applying compaction pressure substantially reduced $\tau_{avg}$, and this effect was most pronounced at greater specimen thicknesses. The results also show that increasing thickness alone without compaction does not reduce acoustic-energy transmission as substantially as the combination of greater thickness and higher compaction pressure.

From a practical perspective, the results confirm that compaction is a key factor in reducing acoustic-energy transmission through the loose textile fraction. The lowest $\tau_{avg}$ values were achieved in the sample with the combination of the greatest thickness, *h_s_* = 7.0 cm, and a compaction pressure of *p* = 500–750 kPa, corresponding to the highest sound-insulation performance among the evaluated configurations.

#### 3.4.2. Relative Transmission Ratio

The relative transmission ratio *RTR_f_*, defined by Equation (6), was used to compare the transmission behavior of specimens made from the loose textile fraction with that of compact textile specimens CT. This parameter expresses the ratio of the transmission coefficients $\tau_{f}$ of two specimens compared at the same frequency *f* and identifies which configuration transmits a greater or smaller fraction of acoustic energy. A value of *RTR_f_* > 1 means that the specimen made from the loose textile fraction transmits more acoustic energy than the compact textile specimen CT and therefore has lower sound-insulation performance. A value of *RTR_f_* < 1 means that the specimen made from the loose textile fraction transmits less acoustic energy than the CT specimen, corresponding to higher sound-insulation performance. A value of *RTR_f_* ≈ 1 indicates comparable transmission behavior of the two material forms.

Table 8 summarizes the statistical characteristics of *RTR_f_* for specimens made from the loose textile fraction at the individual combinations of thickness and compaction pressure, with compact textile specimens CT used as the reference. The minimum, maximum, mean, and standard-deviation values were determined over the frequency range 100–2500 Hz.

The results presented in Table 8 show that *RTR_f_* decreased systematically with increasing compaction pressure, with a more pronounced effect at greater specimen thicknesses. For uncompacted specimens (*p* = 0 kPa), *RTR_f_* was substantially greater than 1 at all thicknesses, indicating higher acoustic-energy transmission than through the reference CT specimens. The greatest difference occurred at *h_s_* = 7 cm, where the mean $RTR_{f}$ was 283.25 and the maximum exceeded 1200. Because the distribution of *RTR_f_* was strongly skewed by several high values at higher frequencies, the median *RTR_f_* of 94.19 provides a more representative measure of the typical difference than the mean value of 283.25. At *p* = 250 kPa, $RTR_{f}$ remained above 1 at *h_s_* = 2 and 4.5 cm, whereas the 7 cm specimens exhibited a transition around $RTR_{f}=1$. At *p* = 500 kPa, transmission was comparable to that of CT at 2 and 4.5 cm but lower at 7 cm. At *p* = 750 kPa, $RTR_{f}$ was below 1 at all thicknesses, with the lowest values observed at 7 cm (0.01–0.17).

These results show that the pressure required to achieve acoustic-energy transmission comparable to or lower than that of CT decreases as specimen thickness increases. At *h_s_* = 2 cm, comparable behavior was achieved at approximately *p* = 500 kPa, whereas at *h_s_* = 7 cm, the transition to more favorable transmission behavior occurred between *p* = 250 and 500 kPa.

**Figure 15:**
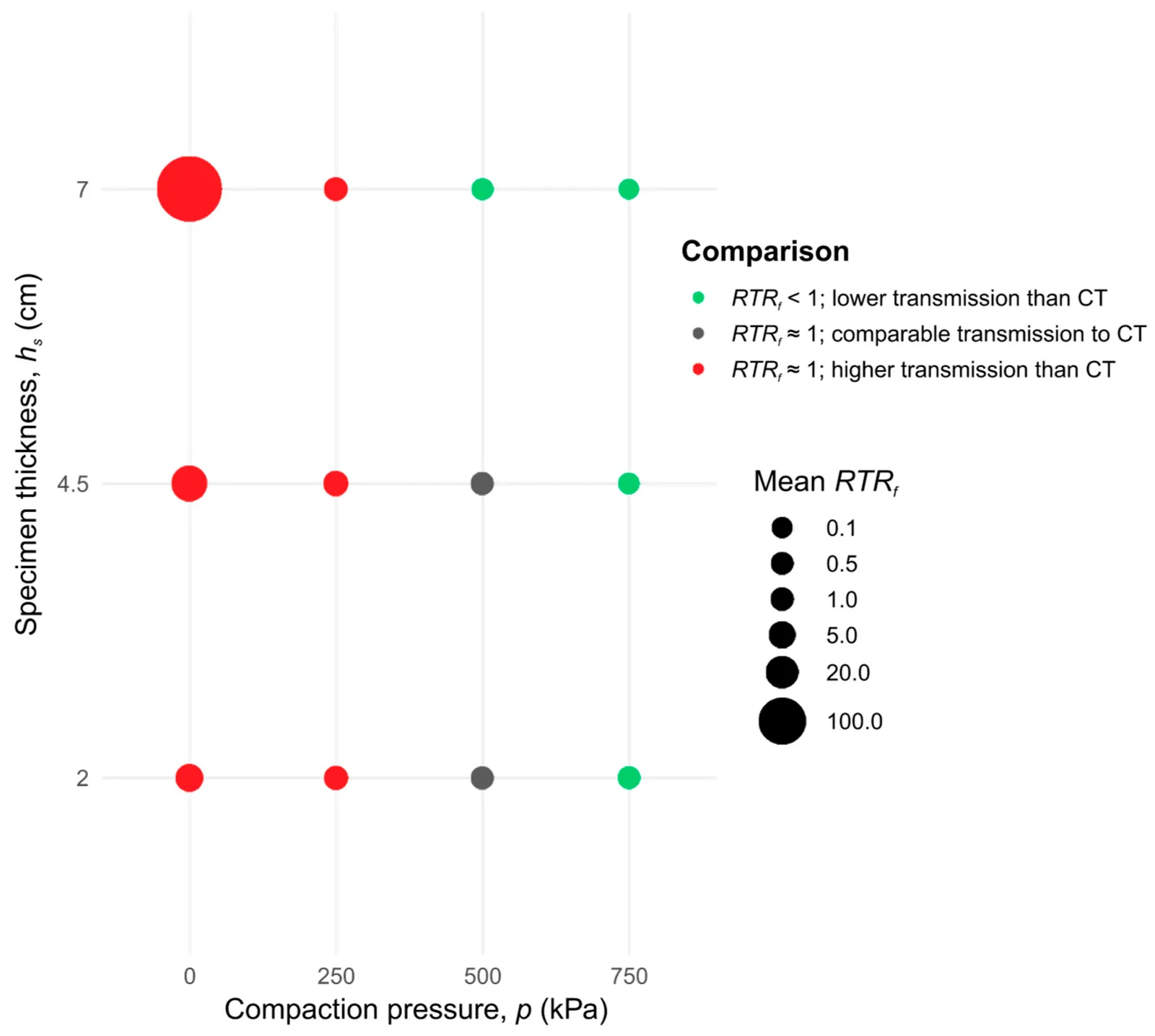
Mean relative transmission ratio, *RTR_f_*, of loose textile fraction specimens compared with compact textile specimens CT.

Figure 15 visually complements Table 8 by showing the change in mean *RTR_f_* as a function of specimen thickness and compaction pressure. The figure shows that the uncompacted specimens L lie in the high-*RTR_f_* region at all thicknesses, confirming their substantially greater acoustic-energy transmission relative to CT. The red dots (*RTR_f_* > 1) indicate cases in which the loose textile fraction transmits more acoustic energy than CT and therefore has poorer sound-insulation properties; the gray dotspoints (*RTR_f_* ≈ 1) indicate comparable transmission behavior; and the green dots (*RTR_f_* < 1) indicate lower acoustic-energy transmission than CT.

Figure 15 shows that the uncompacted specimens L lie in the high-*RTR_f_* region at all thicknesses, confirming their substantially greater acoustic-energy transmission relative to CT. As compaction pressure increases, *RTR_f_* systematically decreases, and the points shift toward the region of comparable or lower transmission. This shift is most pronounced at greater specimen thicknesses, particularly at *h_s_* = 7 cm. Figure 14 therefore confirms that the ability of the loose textile fraction to achieve transmission behavior comparable to or more favorable than that of CT depends on the combination of compaction pressure and specimen thickness. At higher pressures, particularly *p* = 500 and 750 kPa, and at greater thicknesses, the *RTR_f_* values fall below 1, indicating lower acoustic-energy transmission than through the reference CT specimens.

## 4. Discussion

The results of this study show that the sound transmission loss of automotive textile recyclate is influenced primarily by the material configuration—that is, whether the material is an uncompacted loose textile fraction, a compacted loose textile fraction, or a compact textile specimen—as well as by specimen thickness and compaction pressure. For the uncompacted loose textile fraction, increasing specimen thickness alone produced only a slight increase in TL, suggesting that, without compaction, greater thickness was insufficient to substantially reduce acoustic-energy transmission. In contrast, controlled compaction markedly reduced sound transmission, particularly when combined with greater specimen thickness. The observed increase in TL with compaction is consistent with changes generally associated with compression of the fibrous structure in fibrous porous materials [4,6,13,34]. Compaction may alter the arrangement of fibers and textile fragments, the size and distribution of air voids, and the contacts among the individual material components, which may subsequently affect the transmission of acoustic energy through the specimen [4,6,34]. Because porosity, airflow resistance, pore-size distribution, and other parameters characterizing the internal material structure were not directly measured in this study, these relationships should be regarded as a physically justified interpretation of the observed results rather than as experimental confirmation of specific sound-propagation mechanisms.

Previous studies of recycled bulk and fibrous materials have shown that their acoustic properties depend on material form, fraction size, layer thickness, manner of placement, and degree of compaction [37,39,48]. Although the loose textile fraction differs morphologically from bulk materials composed of particles, both are loosely arranged heterogeneous systems whose acoustic behavior is influenced by the arrangement of particles or fibers, internal contacts, and airflow through the pore structure. The present study extends these findings by providing a quantitative evaluation of the transmission loss of a loose textile fraction derived from automotive textile recyclate under controlled changes in compaction pressure and specimen thickness.

In the broader context, research on fibrous, nonwoven, porous, and layered acoustic materials confirms that acoustic performance depends not only on the amount of material or its thickness, but also on internal arrangement, pore structure, layer configuration, and material parameters [49,50,51,52]. The present study builds on these findings by focusing on the transmission loss of automotive textile recyclate rather than on sound absorption.

The dominance of the *p × hs* interaction for the loose-textile-fraction specimens indicates that the effect of compaction on TL depends on material-layer thickness. With increasing compaction pressure, bulk density increased and the arrangement of textile particles and fibers changed in the investigated configurations. In fibrous porous materials, such structural changes may be associated with changes in pore size and arrangement, fiber contacts, and airflow conditions within the material, which affect the transmission and attenuation of acoustic energy [4,6,7,34,53,54]. In contrast, the compact textile specimens (CT) already had a cohesive, compressed, and mechanically bonded fibrous structure stabilized with a PUR binder before measurement and were not further compacted during the experiment. For this material form, the *f × hs* interaction was dominant, meaning that the effect of thickness on TL varied with frequency. Such behavior is physically consistent with frequency-dependent sound propagation and attenuation in a fibrous layer of finite thickness. The difference between the dominant interaction terms of the two models can therefore be associated with the different states and internal arrangements of the two material forms. Because parameters characterizing the internal material structure were not directly measured in this study, these relationships represent a physically justified interpretation of the statistical results rather than direct experimental confirmation of specific sound-propagation mechanisms. From an application perspective, it is important that, within the tested configurations, a suitably compacted loose textile fraction achieved transmission behavior comparable to or more favorable than that of the compact textile specimens. This finding indicates its potential as a material-efficient alternative to industrially manufactured compact, bonded, or pressed panels, particularly when stable material placement can be achieved mechanically without chemical binders. Such an approach could facilitate disassembly, reprocessing, and broader material recovery of textile waste. However, these assumptions must be verified separately, particularly with respect to economic feasibility, environmental benefits, and material stability under real-world conditions.

The experimental design used has an inherent limitation. Because the specimens were prepared at defined thicknesses, compaction pressure could not be varied independently of specimen mass, bulk density, and areal mass. The observed relationships between compaction pressure and TL therefore reflect combined changes in compaction state and the amount of material rather than an experimentally isolated effect of pressure itself. The areal-mass analysis (Section Relationship Between Transmission Loss and Areal Mass) showed that compaction pressure remained statistically associated with *TL_avg_* after accounting for areal mass; however, under the present experimental design, these effects cannot be completely separated. In addition, the compact CT specimens differ from the loose textile fraction not only in bulk density and areal mass, but also in their coherent bonded structure, the presence of a PUR binder, the preparation method, and the measurement mounting. The comparison with CT therefore represents a comparison of specific material configurations and cannot be attributed to a single isolated material characteristic. To unambiguously separate the effect of compaction from the effect of the amount of material, an experiment at constant areal or bulk density would be required.

In acoustic structures, fibrous and porous absorptive materials are often combined with perforated facing elements that provide mechanical protection and stabilization; similar solutions are also used in absorptive noise-control structures [55,56]. Because the perforated element itself can affect the acoustic behavior of the system [56], the effect of using the test cassette and reducing the diameter of the tested specimen was experimentally verified. The control measurement showed a difference of approximately 1–1.5 dB in the individual frequency bands. In addition, all L, C250, C500, and C750 configurations were measured in the same cassette and with the same geometry, thereby maintaining identical conditions for their mutual comparison. The comparison with CT therefore refers to the specific tested configurations.

When interpreting the results, it should be considered that the measurements were performed in an impedance tube under controlled laboratory conditions and over the frequency range 100–2500 Hz. The results therefore primarily provide comparative data for the individual specimen configurations rather than a direct prediction of behavior in actual automotive or building assemblies. TL measurements performed in an impedance tube can be sensitive to the measurement-system configuration and may exhibit greater deviations at certain frequencies [57]. Nevertheless, this approach enabled the systematic comparison of the effects of thickness and compaction pressure on sound transmission through the evaluated material forms.

The variability of the technical repetitions characterizes measurement repeatability for an unchanged specimen. Because the experiment was conducted using a single homogenized batch of automotive textile recyclate and one prepared specimen per experimental configuration, variability among independently prepared specimens and different material batches was not quantified. Evaluation of these sources of variability requires a separate experimental program involving multiple material batches and independently prepared specimens.

Future research should complement the present sound-transmission analysis with direct determination of porosity, airflow resistivity, pore-size distribution, and other parameters characterizing the internal material structure at the individual compaction states. Such data would allow changes in material structure to be linked more precisely to the observed changes in TL and would also provide a suitable basis for physics-based modeling of sound propagation. From a practical perspective, the long-term stability of thickness, bulk density, and material arrangement under vibration, humidity, and repeated loading, as well as the material’s fire-safety and hygienic properties, should also be verified. The economic and environmental benefits of the proposed solution should subsequently be confirmed through a techno-economic analysis and life-cycle assessment relative to conventional bonded or pressed panels. Despite these limitations, the results confirm that an appropriately selected combination of compaction and layer thickness can substantially improve the sound-insulation behavior of the loose textile fraction without requiring its conversion into a compact bonded panel.

## 5. Conclusions

This study experimentally evaluated the sound transmission loss of automotive textile recyclate. Uncompacted and compacted specimens made from the loose textile fraction were compared with compact textile specimens prepared from board insulation material. The results confirmed that the sound-insulation behavior of automotive textile recyclate depends primarily on material form, specimen thickness, compaction pressure, and their interactions. The principal findings of the study can be summarized as follows:

1. The uncompacted loose textile fraction L exhibited the lowest transmission-loss values and the greatest acoustic-energy transmission. Increasing the thickness from *h_s_* = 2 cm to *h_s_* = 7 cm increased the mean $TL_{avg}$ only from 4.20 to 6.27 dB, confirming that thickness alone had only a limited effect on the sound-insulation performance of the uncompacted loose textile fraction.
2. The compaction of the loose textile fraction substantially improved its ability to limit sound transmission. For specimen C750, $TL_{avg}$ increased from 14.98 dB at *h_s_* = 2 cm to 36.34 dB at *h_s_* = 7 cm. The highest sound-insulation performance was achieved for configuration C750-70, that is, at a compaction pressure of *p* = 750 kPa and a thickness of *h_s _*= 7 cm.
3. The combination of greater thickness and higher compaction pressure had a decisive effect on reducing acoustic-energy transmission. The regression model with interaction terms for specimens made from the loose textile fraction had high explanatory power (*R*^2^ = 0.933; adjusted *R*^2^ = 0.930). The most important model term was the interaction between compaction pressure and specimen thickness (*p* × *h_s_*), which had the highest standardized regression coefficient (Std. *β* = 0.764).
4. The comparison using the relative transmission ratio showed that the uncompacted loose textile fraction L transmitted substantially more acoustic energy than the compact textile specimens CT. At *h_s_* = 7 cm, the mean *RTR_f_* for L was 283.25, with a maximum of 1262.99. Increasing the compaction pressure substantially reduced transmission: the mean *RTR_f_* was 0.25 for C500-70 and only *RTR_f_* = 0.08 for C750-70, indicating lower acoustic-energy transmission than through the reference CT specimens.
5. Regression modeling showed that the relative importance of the investigated variables and their interactions differed between the evaluated material forms. For specimens made from the loose textile fraction, the combined effect of compaction pressure and specimen thickness (*p* × *h_s_*) was decisive, whereas for compact textile specimens CT, the interaction between frequency and specimen thickness (*f* × *h_s_*) predominated. The CT model had very high explanatory power (R^2^ = 0.976; adjusted *R*^2^ = 0.975), and the dominant term was the *f* × *h_s_* interaction, with a standardized regression coefficient of *β* = 0.806.

The contribution of this article lies in the systematic comparison of loose, compacted, and compact forms of automotive textile recyclate in terms of sound transmission loss. Most previous studies have focused primarily on finished board, felted, pressed, or nonwoven textile materials. This study therefore expands the existing knowledge by providing experimental data on the less extensively investigated loose textile fraction and its behavior under controlled compaction.

Within the tested configurations, the compacted loose-textile-fraction specimens at pressures of 500 and 750 kPa achieved TL values comparable to or higher than those measured for the reference CT configurations. This finding is particularly important for the potential use of a less-processed form of automotive textile recyclate whose acoustic properties can be deliberately controlled by adjusting the thickness and the degree of compaction. The study thus highlights the potential of secondary textile materials for transportation and technical applications focused on sound insulation and the reduction in acoustic-energy transmission.

## 6. Patents

The test cartridge described in this paper is the subject of patent application No. 50024-2022, “Test Cartridge for Expanding the Possibilities of Measuring Bulk Materials in an Impedance Tube”, which was granted by the Industrial Property Office of the Slovak Republic.

The laboratory device for filling and compaction with digital control described in this article is the subject of published patent application no. 50022-2022, “Device for Measured Filling of Test Cartridges with Bulk Materials”, which was granted by the Industrial Property Office of the Slovak Republic.

## Figures and Tables

**Table 1:** Coding and description of evaluated forms of automotive textile recyclate.

Code	Material Form	State	*p* (kPa)	Description
L	Loose fraction	Uncompacted	0	Irregular mixture of textile particles, fibrous tufts, and loose fibers. Material placed in the test cassette without additional mechanical compaction.
C250	Loose fraction	Compacted	250	The same loose textile fraction as L; after being placed in the test cassette, compacted to the required specimen thickness of *h* = 20, 45, or 70 mm at the corresponding compaction pressure.
C500	Loose fraction	Compacted	500	
C750	Loose fraction	Compacted	750	
CT	Compact specimen	No compaction	–	Circular compact textile specimens prepared from board insulation material made from automotive textile recyclate. The material has a cohesive, compressed fibrous structure.

**Table 2:** Manufacturer-provided material characteristics of the board insulation material used for preparing compact textile specimens.

Parameter	Symbol	Unit	Value
Bulk density	ρ	kg·m^−3^	200
Thermal conductivity	λ	W·m^−1^·K^−1^	0.054
Compressive stress at 10% deformation	σ_10_	kPa	20.3
Tensile strength perpendicular to the plane of the board	σ_t,⊥_	kPa	32.2

**Table 3:** Basic data of specimen configurations used for TL evaluation.

Code	Material Form	State	*d* (mm)	*p* (kPa)	*h* (mm)	*m* (g)	ρ (kg·m^−3^)	*m_s_* (kg.m^−2^)
L-20	Loose fraction	Uncompacted	49	0	20	8.4	223	4.45
L-45	Loose fraction	Uncompacted		0	45	9.2	108	4.88
L-70	Loose fraction	Uncompacted		0	70	17.5	133	9.28
C250-20	Loose fraction	Compacted	49	250	20	11.6	308	6.15
C250-45	Loose fraction	Compacted		250	45	18.8	222	9.97
C250-70	Loose fraction	Compacted		250	70	37.1	281	19.67
C500-20	Loose fraction	Compacted		500	20	13.6	361	7.21
C500-45	Loose fraction	Compacted		500	45	23.1	272	12.25
C500-70	Loose fraction	Compacted		500	70	44.5	337	23.60
C750-20	Loose fraction	Compacted		750	20	14.9	395	7.90
C750-45	Loose fraction	Compacted		750	45	28.8	339	15.27
C750-70	Loose fraction	Compacted		750	70	51.8	392	27.47
CT-20	Compact specimen	No compaction	60	–	20	10.0	177	3.54
CT-45	Compact specimen	No compaction		–	45	18.3	144	6.47
CT-70	Compact specimen	No compaction		–	70	30.0	152	10.61

L—uncompacted loose textile fraction; C250, C500 and C750—loose textile fraction compacted at 250, 500 and 750 kPa, respectively; CT—compact textile specimen; *p*—compaction pressure; *h*—specimen thickness; *d*—specimen diameter used for volume calculation; *m*—specimen mass; ρ—bulk density; *m_s_*—specimen areal mass (kg·m^−2^); calculated as *m*/*A*, where *m* is the specimen mass and *A* is its cross-sectional area.

**Table 4:** Overview of symbols, sample codes and parameters used in the study.

Symbol/Code	Description
*k*	specimen code used in the experiment (e.g., L-20, C250-45, CT-70)
L	uncompacted loose textile fraction (*p* = 0 kPa)
C250	loose textile fraction compacted at a nominal compaction pressure of *p* = 250 kPa
C500	loose textile fraction compacted at a nominal compaction pressure of *p* = 500 kPa
C750	loose textile fraction compacted at a nominal compaction pressure of *p* = 750 kPa
CT	compact textile specimens prepared from board insulation material
*h*	specimen thickness used in the configuration codes (mm), *h* = 20, 45, 70 mm
*hs*	specimen thickness used to interpret the results (cm), *h_s_* = 2, 4.5, 7 cm; converted to meters for regression modeling
*p*	compaction pressure (kPa), *p* = 0, 250, 500, 750 kPa
ρ	bulk density (kg·m^−3^)
*m_s_*	areal mass (kg·m^−2^)
*f*	frequency (Hz), frequency range 100–2500 Hz
TL	transmission loss (dB)
*τ_f_*	transmission coefficient at frequency f (–)
*τ*_avg_	average transmission coefficient (–)
*TL_f_*	transmission loss at frequency f (dB)
*TL*_avg_	average transmission loss (dB)
*RTR_f_*	relative transmission ratio at frequency f (–)

**Table 5:** Regression models for transmission loss TL.

Parameter	Coefficient	Std. *β*	Std. Error	OLS *p*-Value	Interpretation	VIF
**Model I:** Loose textile fraction specimens main effects (*n* = 180, *df*_res_ = 176; *F*(3, 176) = 289.1, *p*-value < 2 × 10 − 16), *R*^2^ = 0.831, adjusted *R*^2^ = 0.828						
Intercept	−1.074 × 101	–	1.125	<10^−16^	***	
*f*	4.621 × 10^−3^	+0.271	5 × 10^−4^	1.7 × 10^−15^	***	1.00
*p*	2.923 × 10^−2^	+0.667	0.001	<10^−16^	***	1.00
*h_s_*	3.331 × 102	+0.552	18.67	<10^−16^	***	1.00
**Model II:** Loose textile fraction specimens main effects and interactions (*n* = 180, *df*_res_ = 173; *F*(6, 173) = 399.0, *p*-value < 2 × 10^−16^), *R*^2^ = 0.933, adjusted *R*^2^ = 0.930						
Intercept	4.802	–	1.224	1.2 × 10^−4^	***	
*f*	−2.41 × 10^−3^	−0.141	9 × 10^−4^	0.010	*	7.62
*p*	−9.2 × 10^−5^	−0.002	2 × 10^−3^	0.968	not significant	7.03
*h_s_*	7.508	+0.012	24.11	0.756	not significant	4.10
*f* × *p*	3.5872 × 10^−6^	+0.118	1 × 10^−6^	0.003	**	4.01
*f* × *h_s_*	0.128	+0.406	0.017	9 × 10^−13^	***	7.13
*p* × *h_s_*	0.593	+0.764	0.043	<10^−16^	***	7.78
**Model III:** Compact textile specimens (*n* = 45, *df*_res_ = 41; *F*(3, 41) = 562.5, *p*-value < 2 × 10^−16^), *R*^2^ = 0.976, adjusted *R*^2^ = 0.975						
Intercept	8.482	–	0.668	<10^−16^	***	
*f*	−2.12 × 10^−3^	−0.198	6.1 × 10^−4^	0.002	**	5.86
*h_s_*	1.81 × 10^2^	+0.479	13.52	<10^−16^	***	2.21
*f* × *h_s_*	1.601 × 10^−1^	+0.806	0.013	1 × 10^−15^	***	7.07

Std. *β*—standardized regression coefficient; Std. Error—standard error; VIF—variance inflation factor; OLS—ordinary least squares; model *p*-value—overall statistical significance of the regression model; *p*-value in parameter rows—probability value used to assess the statistical significance of individual parameters at the *α* = 0.05 significance level. Significance levels are denoted as follows: *** highly statistically significant parameter or variable (*p*-value < 0.001), ** moderately statistically significant parameter (*p*-value < 0.01), * statistically significant parameter (*p*-value < 0.05); “not significant” indicates *p*-value ≥ 0.05. VIF values were interpreted as follows: VIF < 5 indicates low to acceptable multicollinearity, 5 ≤ VIF < 10 indicates moderate multicollinearity and VIF ≥ 10 indicates high multicollinearity. The symbol *f* denotes frequency; *p* denotes compaction pressure; *h_s_* denotes specimen thickness. For regression modelling, *h_s_* values were entered in meters, i.e., 0.020, 0.045 and 0.070 m; *n*—number of observations (band-wise mean TL values, each the mean of five repeated measurements; 12 configurations × 15 one-third-octave bands for Models I and II, 3 CT configurations × 15 one-third-octave bands for Model III); *df*_res_—residual degrees of freedom; *F*—F-statistic of the overall model test.

**Table 6:** Leave-one-specimen-out cross-validation results for the regression models.

Model	*R* ^2^	*R* ^2^ *cv*	RMSE (dB)	MAE (dB)	Bias (dB)	*r*
Model I—loose textile fraction	0.831	0.688	6.85	5.03	–0.15	0.835
Model II—loose textile fraction	0.933	0.836	4.97	3.57	–0.70	0.920
Model III—compact textile specimens	0.976	–	–	–	–	–

*R*^2^—coefficient of determination of the model developed from the full experimental dataset; *R*^2^_CV_—cross-validated coefficient of determination obtained from leave-one-specimen-out predictions; RMSE—root mean square error; MAE—mean absolute error; bias—mean prediction error; *r*—Pearson correlation coefficient between the measured and cross-validated predicted TL values. Model III was not subjected to specimen-level cross-validation because only three physical CT specimens were available.

**Table 7:** Interpretation of variable importance based on standardized regression coefficients.

Parameter	Std. *β*	Direction/Effect	Interpretation
**Model I:** Loose textile fraction specimens, main effects			
*f*	+0.271	↑ weak positive	Frequency has a slight positive effect on TL. Transmission loss increases slightly with increasing frequency.
*p*	+0.667	↑↑ strong positive	Compaction pressure has a strong positive effect on TL. Transmission loss increases substantially with increasing compaction pressure.
*h_s_*	+0.552	↑↑ strong positive	Specimen thickness has a substantial positive effect on TL. Transmission loss increases with increasing specimen thickness.
**Model II:** Loose textile fraction specimens, main effects and interactions			
*f*	−0.141	conditional main effect	Conditional effect of frequency at *p* = 0 kPa and *h_s_* = 0 m (outside the tested range); not interpretable as an overall frequency effect. Within the tested range, TL increases with frequency for all configurations.
*p*	−0.002	conditional main effect	Conditional effect of compaction pressure at *f* = 0 Hz and *h_s_* = 0 m (outside the tested range); not interpretable as an overall pressure effect. Its non-significance does not imply the absence of a pressure-related change in TL within the tested range.
*h_s_*	+0.012	conditional main effect	Conditional effect of specimen thickness at *f* = 0 Hz and *p* = 0 kPa; *f* = 0 Hz is outside the tested range. Therefore, the coefficient is not interpretable as an overall thickness effect.
*f* × *p*	+0.118	↑ weak positive interaction	Positive interaction indicating that the association between frequency and TL becomes stronger with increasing compaction pressure (see Figure 9b); marginally significant with robust standard errors (*p* = 0.051).
*f* × *h_s_*	+0.406	↑↑ strong positive interaction	Positive interaction indicating that the association between frequency and TL becomes stronger with increasing specimen thickness (see Figure 10a).
***p*** **×** ***h_s_***	**+0.764**	**↑↑↑ strongest positive interaction**	Strongest interaction in Model II; the association between compaction pressure and TL becomes stronger with increasing specimen thickness (see Figure 9a).
**Model III:** Compact textile specimens			
*f*	−0.198	conditional main effect	Conditional effect of frequency at *h_s_* = 0 m (outside the tested range); the negative coefficient does not imply decreasing TL with frequency at the tested specimen thicknesses.
*h_s_*	+0.479	conditional main effect	Conditional effect of specimen thickness at *f* = 0 Hz (outside the tested range); not interpretable as an overall thickness effect. The thickness-related change in TL depends on frequency
***f*** **×** ***h_s_***	**+0.806**	**↑↑↑ strongest positive interaction**	Strongest interaction in Model III; the association between frequency and TL becomes stronger with increasing specimen thickness (see Figure 10b).

Std. *β*—standardized regression coefficient; *f*—frequency; *p*—compaction pressure; *h_s_*—specimen thickness; ↑—indicates a weak positive effect on *TL*; ↑↑—indicates a strong positive effect on *TL*; ↑↑↑—indicates a strongest positive effect on *TL*. In Model I, arrows indicate the direction and relative magnitude of the associations with TL. In Models II and III, arrows are used for interaction terms, whereas the main-effect coefficients are interpreted conditionally at zero values of the interacting variables and not as overall effects. The number of arrows reflects the relative magnitude of the standardized coefficient within the given model.

**Table 8:** Relative transmission ratio *RTR_f_* of the loose textile fraction compared with compact textile specimens CT.

*h_s_* (cm)	*p* (kPa)	Min	Max	Mean	Median	SD	*RTR_f_* vs. CT	Interpretation
2	0	3.30	11.74	7.67	7.90	2.71	*RTR_f_* > 1	high transmission
2	250	0.85	2.18	1.76	1.82	0.37	*RTR_f_* > 1	higher transmission
2	500	0.54	1.15	0.92	0.96	0.17	*RTR_f_* ≈ 1	comparable transmission
2	750	0.37	0.74	0.62	0.65	0.10	*RTR_f_* < 1	lower transmission
4.5	0	12.41	74.10	33.74	23.82	19.44	*RTR_f_* > 1	very high transmission
4.5	250	1.86	4.40	3.11	2.96	0.66	*RTR_f_* > 1	high transmission
4.5	500	0.42	1.31	0.95	1.01	0.29	*RTR_f_* ≈ 1	comparable transmission
4.5	750	0.03	0.19	0.13	0.15	0.04	*RTR_f_* < 1	low transmission
7	0	53.06	1262.99	283.25	94.19	369.36	*RTR_f_* > 1	very high transmission
7	250	0.57	2.52	1.45	1.39	0.51	transition	transition range
7	500	0.03	0.50	0.25	0.24	0.15	*RTR_f_* < 1	low transmission
7	750	0.01	0.17	0.08	0.09	0.05	*RTR_f_* < 1	low transmission

CT—compact textile specimens used as reference specimens; Min, Max, Mean, Median, and SD describe the variation in *RTR_f_* across the 15 one-third-octave bands between 100 and 2500 Hz (spectral variability); they do not represent measurement uncertainty. For the uncompacted specimens, the distribution of *RTR_f_* is strongly skewed, so the median is more representative than the mean. The category *RTR_f_* > 1 indicates that the loose textile fraction transmits more acoustic energy than CT, *RTR_f_* ≈ 1 indicates comparable transmission behavior, and *RTR_f_* < 1 indicates lower acoustic-energy transmission than CT.

## Data Availability

The original contributions presented in this study are included in the article. Further inquiries can be directed to the corresponding author.
